# Differences in multidimensional phenotype of 2 joint pain models link early weight-bearing deficit to late depressive-like behavior in male mice

**DOI:** 10.1097/PR9.0000000000001213

**Published:** 2024-11-20

**Authors:** Sara Hestehave, Roxana Florea, Alexander J.H. Fedorec, Maria Jevic, Lucile Mercy, Annia Wright, Oakley B. Morgan, Laurence A. Brown, Stuart N. Peirson, Sandrine M. Géranton

**Affiliations:** a Department of Cell & Developmental Biology, University College London, London, United Kingdom. Hestehave is now with the Department of Experimental Medicine, University of Copenhagen, Copenhagen N, Denmark; b Research IT, University of Oxford, Oxford, United Kingdom; c Nuffield Department of Clinical Neuroscience, University of Oxford, Oxford, United Kingdom

**Keywords:** Joint pain, Prediction, Weight bearing, Hypersensitivity, Affective symptoms, Long-term outcome

## Abstract

**Introduction:**

Chronic pain is a hallmark of joint diseases. Although these conditions are often accompanied by negative affective symptoms including depression and anxiety, these comorbidities are rarely studied simultaneously in preclinical models where they are poorly characterised. Moreover, how affective symptoms relate to the more obvious sensory and functional symptoms of joint diseases is not well understood. Here, we have addressed these gaps in knowledge.

**Methods:**

We used 2 preclinical models of joint pain in male mice and an array of behavioural and molecular assays to fully characterise functional deficits, mechanical hypersensitivity, affective symptoms, and nociceptive signaling in joint pain, as well as investigate their relationship.

**Results:**

Ankle joint inflammation and knee osteoarthritis induced mechanical hypersensitivity that lasted at least 3 months and that was not different between the 2 models on most days. However, the models presented with markedly different weight-bearing deficits, molecular profiles, and affective outcomes. Specifically, only the model of knee osteoarthritis was accompanied by an increase in negative affective behaviors, including early changes in circadian patterns, persistent cognitive impairments, and late development of depressive-like behavior. Importantly, the early weight-bearing deficit strongly correlated with the emotional profiles and the hypersensitivity at 3 months, suggesting that early objective functional measures may be used as predictors of long-term affective symptoms and pain.

**Conclusion:**

The predictive value of early weight-bearing deficit could prove useful in the clinical setting for adapted therapeutic approaches for the prevention of emotional comorbidities and better pain management for patients with joint pain.

## Abstract 2

Supplemental Digital Content is Available in the Text.

Using preclinical models, we demonstrate that early functional measures may be used as predictors of long-term affective symptoms and pain in joint disease.

## 1. Introduction

The prevalence of clinically significant anxiety and depression with chronic joint pain is at least 41%,^4^ with patients often experiencing a range of negative affective symptoms that positively correlate with the pain severity.^51^ Although the sensory aspects of joint pain have been extensively studied in animal models, associated mood-related disorders are not often enough assessed in preclinical research. As a result, it remains uncertain whether joint pain models commonly used in preclinical research possess sufficient translational value to enhance the understanding of human diseases and the development of effective therapies. Moreover, as sensory, functional, and affective outcomes of joint pain have rarely been assessed simultaneously, the relationship between these symptoms remains poorly understood. However, a better understanding of how the early pain-related signs of joint disease, such as gait changes, and the long-term affective outcomes and hypersensitivity may be connected could support better clinical management and help with the prevention of significant comorbidities by early intervention.

To address these gaps in knowledge, we have used 2 well-described models of joint pain with fast onset of mechanical hypersensitivity that are often used in preclinical studies. We used the model of complete Freund adjuvant (CFA)-induced ankle joint inflammation^25,38^ and the monoiodoacetate (MIA) model of knee osteoarthritis.^44,45,52^ Intra-articular CFA injection leads to the infiltration of inflammatory cells and synovial hypertrophy and generates robust and long-lasting pain-like behaviours.^25^ The MIA model induces rapid disease progression in the knee. Monoiodoacetate inhibits the glycolytic pathway causing rapid and widespread chondrocyte death, extensive neovascularization, subchondral bone necrosis, and collapse, as well as profound and prolonged inflammation and pain-related symptoms responsive to conventional pain-relieving therapies.^17,19,45^

Through comprehensive characterization of these models side-by-side, we report that both models presented with subtle differences in sensory profiles across the 3 months of monitoring, but significant differences in weight-bearing deficits and emotional behaviors that unexpectedly only developed after MIA. Importantly, depression-like behaviour that developed 3 months after the onset of the joint pain correlated with weight-bearing deficits measured at the early stage of the disease state. Our results suggest that early functional outcomes of joint disease reflect the overall pain burden and could be used to predict the likelihood to develop emotional comorbidities.

## 2. Methods and materials

Extended materials and methods can be found in supplementary materials, http://links.lww.com/PR9/A264.

### 2.1. Animals and housing

Adult male mice (C57Bl/6J; Charles River, United Kingdom) were housed in temperature- and light-controlled environment with ad libitum provision of food and water. All experiments were performed under the Home Office License P8F6ECC28, and all efforts were made to minimize animal suffering and to reduce the number of animals used (UK Animal Act, 1986).

### 2.2. Study design

The study was divided into cohorts/studies including animals from all experimental groups (CFA vs MIA vs control, n = 4–7), each designed to test a selection of parameters, and terminated at different time points for tissue collection. Therefore, group sizes were variable throughout the study and are presented as a range.

### 2.3. Experimental procedures

#### 2.3.1. Induction of injury

Model induction was performed under anaesthesia and as previously reported for the CFA model of tibio-tarsal joint inflammation,^37^ and the monoiodoacetate arthritis model in the left knee joint.^45^ Control animals were exposed to anaesthesia only. Inflammation was measured by joint circumference (JC) as previously.^5,6^

#### 2.3.2. Behavioural testing

Behavioural testing was always performed in randomized order and by the same female experimenter. For all methods, see full protocols in supplementary material, http://links.lww.com/PR9/A264.

##### 2.3.2.1. Mechanical allodynia

Low-intensity mechanical sensitivity on the ipsilateral paw was assessed using von Frey (VF) monofilaments. The threshold was determined by using the Dixon up–down method, as described by Chaplan and colleagues.^13,18,38^

#### 2.3.2.2. Affective-motivational behaviour

In addition to assessing pure reflexive sensory threshold to mechanical allodynia (VF), the protocol originally described by Corder et al.^14^ was adopted and modified to assess the affective responses displayed after stimulation with 3 selected filaments (low, 0.04 g; medium, 0.16 g; high, 1.0 g).^36^

##### 2.3.2.3. Cold allodynia (acetone drop test)

The duration of pain-like behaviors was assessed for 30 seconds after the application of a drop of acetone (acetone drop test [ADT]) to the plantar surface of the paw.

##### 2.3.2.4. Functional impairment—weight bearing

Static weight bearing (WB) distribution across the hind legs was assessed similarly to previously described^33^ using a Bioseb Incapacitance Test (Bioseb, Vitrolles, France).

##### 2.3.2.5. Catwalk gait analysis

Analysis of voluntary movement and gait pattern was performed using the Catwalk XT 10.0 system (Noldus Information Technology BV, NL^53^) and based on our previous experience.^27^

##### 2.3.2.6. Sucrose preference test

To assess depressive-like behaviour, the sucrose preference test (SPT) was included as a measure of anhedonia.^55^ Preference for a 1% sucrose solution vs normal water was assessed overnight in the homecage environment, without prior food- or water-restriction^7^.

##### 2.3.2.7. Anxiety-like behavior (elevated plus maze and open field test)

To assess anxiety-like behaviour, we used 5-minute tests in an elevated plus maze (EPM),^27,28^ and an open field test (OFT),^26^ with behavior tracked using EthoVision XT14 (Noldus Information Technology).

##### 2.3.2.8. Novel object recognition

The novel object recognition (NOR) test was performed similarly to previously described,^8,26,39^ with some modifications. In short, the protocol consisted of a 10-minute habituation to an arena with 2 similar objects, and 3 hours later, a 5-minute test with a novel object replacing one of the previous objects. The proportion of time spent exploring the objects was assessed by calculating a proportion of time exploring the novel object using the following formula:

$$Discrimination\;index=\frac{\text{Time}\;\text{spent}\;\text{exploring}\;\text{novel}-\text{familiar}\;\text{object}}{\text{Time}\;\text{spent}\;\text{exploring}\;\text{novel}+\text{familiar}\;\text{onject}}.$$

##### 2.3.2.9. Sleep/activity pattern

To measure undisturbed activity in the home cages, we used noninvasive passive infrared motion sensors as before^11^ and monitored single-housed animals for 7 days postinjection. Home cage mouse activity was tracked with measurements taken every 10 seconds across multi-day periods. Sleep was defined as periods in which no activity was measured for 40 seconds or more based on our previous work that detected an almost complete correlation (*r* > 0.95) between electroencephalogram (EEG)-recorded sleep and motion sensor–recorded inactivity for >40 seconds.^11^ Activity and sleep data were smoothed by calculating the mean in 10-minute bins. Several summary statistics of circadian disruption were calculated for individual.^10^

#### 2.3.3. Immunohistochemistry

Mice were perfused transcardially (heparinized saline followed by 4% paraformaldehyde), and the tissue was cut at 40-μm thickness for immunohistochemistry. Section from the lumbar spinal cord was incubated with c-Fos, calcitonin gene–related peptide (CGRP), IBA1, and glial fibrillary acidic protein (GFAP) antibodies, whereas doublecortin (DCX) was assessed in hippocampal sections. Full description of immunohistochemistry and microscopy are available in supplementary material, http://links.lww.com/PR9/A264.

#### 2.3.4. Data and statistical analysis

All statistical tests were performed in IBM SPSS Statistic Program (vers.26) or GraphPad Prism (vers.9), and *P* < 0.05 was considered statistically significant. All details on statistical analysis, factors tested, and significant outcomes can be found in supplementary material and Tables S1 and S2, http://links.lww.com/PR9/A264, and only selected outcomes are presented in the main text.

## 3. Results

### 3.1. Complete Freund adjuvant and monoiodoacetate induce functional deficit and persistent hypersensitivity that last at least 3 months

**Figure 1.**
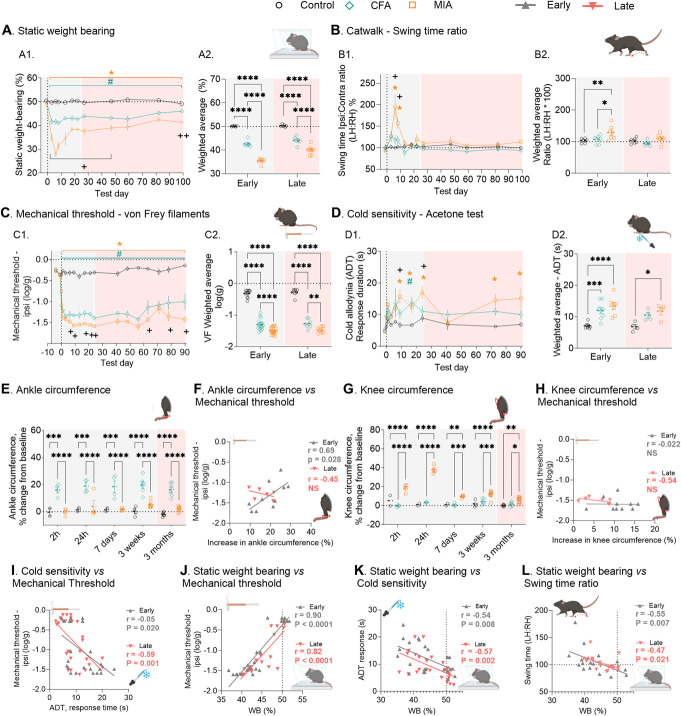
CFA and MIA induce persistent sensory and functional deficit for at least 3 months. (A1, 2) Functional deficits, assessed using static weight-bearing distribution (N = 6/6/7). (B1, 2) Functional deficit, assessed by swing time ratio with the Catwalk gait analysis (N = 6/6/7). (C1, 2) Mechanical hypersensitivity, assessed using Von Frey filaments (N = 4–18). (D1, 2) Cold allodynia, determined using the acetone drop test (ADT) (N = 4–8). (E) Ankle circumference (N = 2–7). (F) Correlation between ankle circumference and mechanical hypersensitivity in CFA-injured animals. (G) Knee circumference. (H) Correlation between knee circumference and mechanical hypersensitivity in MIA-injured animals. (I) Correlation between mechanical threshold and cold sensitivity. (J) Correlation between mechanical threshold and static weight bearing distribution (WB). (K) Correlation between static weight-bearing distribution (WB) and cold sensitivity (ADT). (L) Correlation between swing time ratio and static weight-bearing distribution (WB). Early, day 1 to 25; late, day 26 to 90. Data presented as individual time points or mean ± SEM. Posttest in time-course figures (A1, B1, C1, D1): #*P* < 0.05, CFA vs control; *P < 0.05, MIA vs control, +*P* < 0.05 CFA vs MIA, as determined using Tukey multiple comparison test. Full analysis outcome in Supplementary Table S1, http://links.lww.com/PR9/A264. For scatterplot figures (A2, B2, C2, D2, E, G), posttests between injury groups are displayed using connecting lines: *P < 0.05, **P < 0.01, ***P < 0.001, ****P < 0.0001, as determined using Tukey posttest. CFA, complete Freund adjuvant; MIA, monoiodoacetate.

Static bodyweight distribution is a surrogate measure for the joint pain associated with weight-bearing and has been the key outcome measure for unilateral joint pain models for decades.^9^ Here, we detected clear and prolonged weight-bearing (WB) asymmetry after CFA and MIA (Fig. 1A1). Because of early striking differences between the 2 models, we split the data between an early (day 0 to day 25) and a late phase (day 26 to day 90), as previously reported^57^ (Fig. 1A2). The MIA model showed a strong WB impairment in the early phase (mean ± SEM: 35.4 ± 0.4%), after which it stabilized (40.0 ± 0.8%), whereas CFA produced a more stable deficit across the duration of the observations (early: 42.7 ± 0.6%, late: 44.1 ± 0.7%) (Fig. 1A). Overall, the MIA model caused a higher degree of weight-bearing asymmetry than the CFA model.

A full Catwalk gait analysis confirmed the biphasic nature of the deficit in the MIA model and detected early gait changes after MIA on parameters related to Swing Time (Fig. 1B1, 2, Early: MIA: 128.8 ± 8.3%, control: 103.2 ± 3.1, CFA: 106.6 ± 5.7), Duty Cycle, Stride Length, Single Stance and Print Position (Fig. S1A-D, http://links.lww.com/PR9/A264), with correlations with static WB at peak Day 8 and the 3-week time point (Fig. S1E, F, http://links.lww.com/PR9/A264). Later, the dynamic gait changes were relatively mild and the gait of injured animals were very rarely different from controls (Late: MIA: 109.5 ± 5.6%, CFA: 94.8 ± 3.4%, control: 102.7 ± 3.6%, Fig. 1B1, 2, Fig. S1G, http://links.lww.com/PR9/A264).

To characterise the sensory phenotype, we first measured mechanical thresholds using von Frey (VF) monofilaments. Six hours after injection, only the CFA-treated animals showed allodynia, but from day 1 to day 90, both models produced significant mechanical allodynia (*P* < 0.05–0.0001), with the MIA group significantly more sensitive than CFA on only a few individual days (Fig. 1C1, 2).

Cold allodynia is a significant hallmark of joint diseases and we explored the response to cold using the ADT. Both models induced significant but highly dynamic cold allodynia that was more prominent after MIA in the early phase and absent after CFA in the late phase (Fig. 1D1, 2).

None of the models produced differences in bodyweight compared with control animals (Fig.S1H, http://links.lww.com/PR9/A264). CFA–induced swelling of the ankle was stable from 2 hours after the injection for up to 90 days (Fig. 1E), but surprisingly, there was an inverse relationship between ankle swelling and mechanical threshold during the early phase (day 7 and 25 combined) (Fig. 1F). After a steep increase, the MIA-induced swelling of the knee nearly returned to baseline by day 7 (Fig. 1G), and there was no correlation between knee swelling and mechanical threshold (Fig. 1H).

We finally explored the connection between the sensory and functional outcomes and found a significant correlation between measures recorded on a given animal on the same day at both the early (3 weeks) and the late phase (3 months) of the joint pain development between mechanical thresholds and both cold allodynia (Fig. 1I) and weight bearing (Fig. 1J), between cold allodynia and weight bearing (Fig. 1K), and between dynamic gait and static weight bearing (Fig. 1L).

### 3.2. Complete Freund adjuvant and monoiodoacetate induce different patterns of neuronal and glia activation in the spinal cord

**Figure 2.**
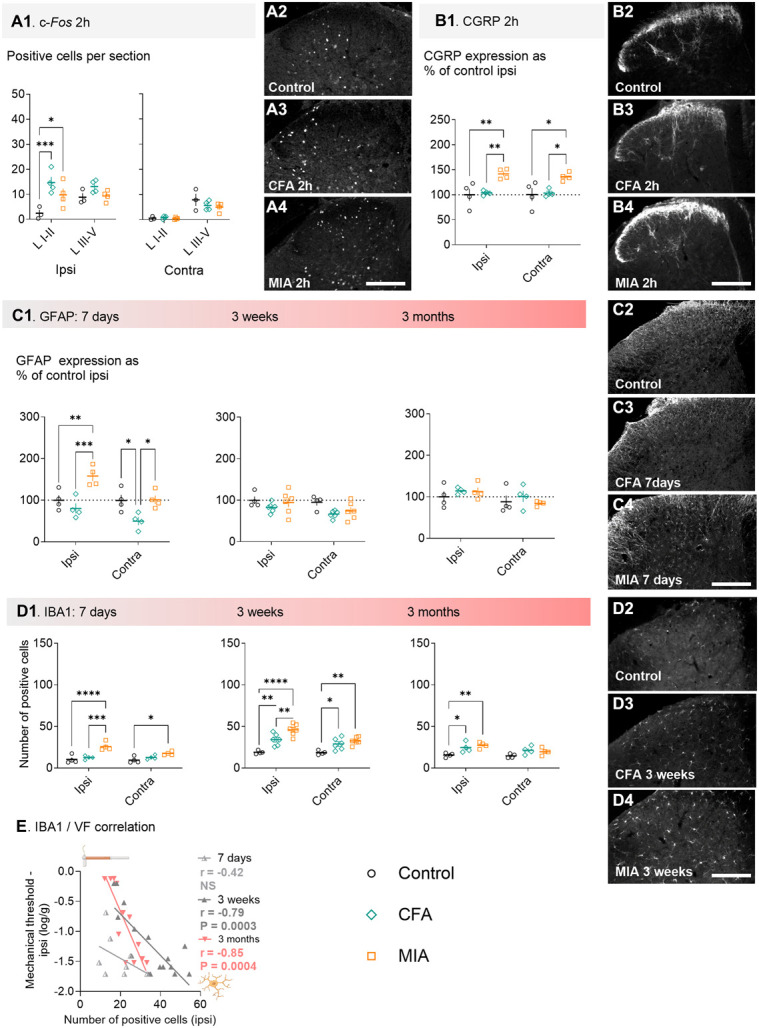
CFA and MIA induce different patterns of neuronal and glia activation in the spinal cord. (A) c-Fos expression 2 hours after the injection of CFA and MIA. Representative picture of c-Fos expression in control (A2), CFA (A3), and MIA (A4) mice in the ipsilateral superficial dorsal horn 2 hours after injection or brief anesthesia for the control group. (B) CGRP expression 2 hours after model induction. Representative picture of typical CGRP expression in control (B2), CFA (B3), and MIA (B4) mice in the ipsilateral superficial dorsal horn 2 hours after injection or brief anesthesia for the control group. (C) glial fibrillary acidic protein (GFAP) expression 7 days, 3 weeks, and 3 months after model induction. Representative picture of typical GFAP expression in control (C2), CFA (C3), and MIA (C4) mice in the ipsilateral superficial dorsal horn 7 days after injection or brief anesthesia for the control group. (D) IBA1 expression 7 days, 3 weeks, and 3 months after model induction. Representative picture of typical IBA1 expression in control (D2), CFA (D3), and MIA (D4) mice in the ipsilateral superficial dorsal horn 3 weeks after injection or brief anesthesia for the control group. (E) IBA1 expression in the superficial dorsal horn correlates with mechanical thresholds (VF) at 3 weeks and 3 months after model induction. Ipsi = ipsilateral to injection; Contra = contralateral to injection. LI-II = laminae I and II; LIII-V = laminae III to V. scale bar = 100 µm; Posttest comparison of injury groups; N = 3 to 6 per treatment group; *P < 0.05, **P < 0.01, ***P < 0.001, ****P < 0.0001, determined using Tukey multiple comparison test. CFA, complete Freund adjuvant; CGRP, calcitonin gene–related peptide; MIA, monoiodoacetate.

We next looked at molecular signaling in the superficial dorsal horn that could underlie the differences in sensory and functional profile observed. Using immunohistochemistry, we found that CFA and MIA induced similar pattern of expression of the immediate early gene c-Fos in the superficial dorsal horn within 2 hours of injection, indicating similar degree of nociceptive input. However, we observed greater levels of the CGRP after MIA than after CFA and control (Fig. 2A,B). This suggested a greater and widespread inflammatory component early in the MIA model.

We next looked at the activation of nonneuronal cells known to contribute to the maintenance of hypersensitive states and occurring later than neuronal activation. Compared with CFA, MIA induced a greater expression of the astrocytic marker GFAP at 7 days (Fig. 2C), as quantified by the overall staining intensity across laminae LI to LII. Both models induced significant upregulation of the microglia marker IBA1 from 7 days to 3 months, as quantified by individual cell counts. However, there was a greater upregulation after MIA when compared with CFA at both 7 days and 3 weeks (Fig. 2D), suggesting a strong neuropathic component with the MIA model. Finally, we found that only IBA1 expression at 3 weeks and 3 months correlated with the degree of allodynia (Fig. 2E).

### 3.3. Activity and sleep patterns in the early phase of the persistent pain states are more disrupted in the monoiodoacetate than in the complete Freund adjuvant model

**Figure 3.**
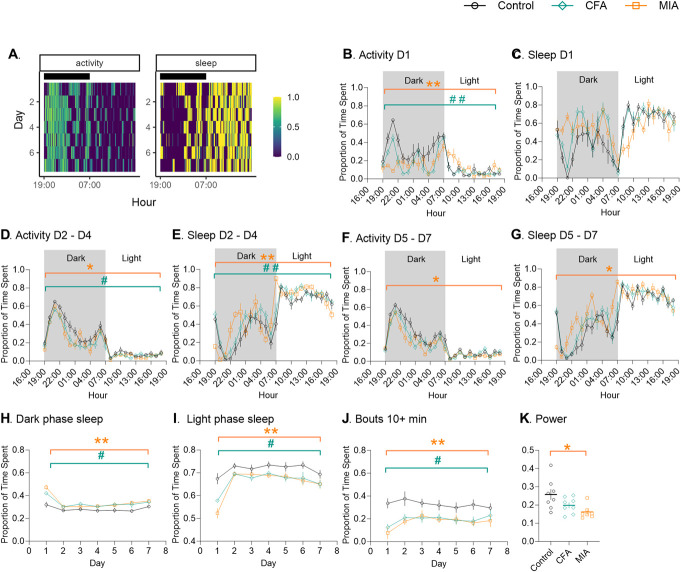
Sleep and activity patterns are more sensitive to MIA than CFA. (A) Representative activity and sleep patterns of a control mouse over 7 days of recordings, using 10-minute bins. (B–G) The 24-hour activity/sleep plots using 1-hour bins. D1 = day 1 after CFA/MIA injection. D2-D4 = average of day 2 to day 4 after CFA/MIA injection. D5-D7 = average of day 5 to day 7 after CFA/MIA injection. (H–J) Seven-day plots using 24-hour bins; (H) Proportion of sleep during the dark period. (I) Proportion of sleep during the light period. (J) Activity bouts of >10 minutes during the dark period. (K) A comparison of power of the 24-hour activity cycle calculated across 7 days. The power of a periodogram (periodogram shown in Fig. S2G, http://links.lww.com/PR9/A264) provides a measure of the strength and regularity of the underlying rhythm, with higher values indicating robust rhythms. In circadian disruption—where rhythms are typically less robust—periodogram power is expected to be reduced, which may indicate the absence of a significant circadian rhythm. (B–J) Data show mean ± SEM. (K) Single data points. N = 8/8/7, control/CFA/MIA; #*P* < 0.05, ##*P* < 0.01, CFA vs control; *P < 0.05, **P < 0.05, MIA vs control, determined using Tukey post hoc analysis after repeated-measures 2-way ANOVA. CFA, complete Freund adjuvant; MIA, monoiodoacetate.

Because the differences in functional outcomes between the 2 models were more prominent in the very early stages of the joint disease, we looked at the activity and sleep patterns in the first week after model induction. Home-cage activity and sleep were tracked immediately after injury, using a system of passive infrared motion sensors^11^ (Fig. 3A).

The activity and sleep patterns were significantly disrupted for the first 24 hours after the CFA and MIA injections and did not resemble that of healthy mice (Fig. 3B,C). However, injured mice recovered rapidly and displayed general patterns of activity and sleep that were overall similar to control mice, but with a significant reduction in total activity and increase in total sleep between day 2 and day 4 after model induction (Fig. 3D,E). Between day 5 and day 7, only the MIA-injured animals remained different from controls (Fig. 3F,G).

Several summary statistics of circadian disruption^10^ suggested that animals recovered rapidly after model induction (Fig. S2, http://links.lww.com/PR9/A264), whereas others indicated longer lasting changes. Injured animals showed a significant increase in dark-phase sleep, a measure of the proportion of daily sleep that occurs when animals are expected to be active compared with control animals over a 7-day period (Fig. 3H). In contrast, light phase sleep was decreased (Fig. 3I), suggesting a shift in sleep. The greatest effect of injury was observed on the number of long bouts of activity that animals engaged with. A bout is classified as a period of sustained activity above 10% of the mean activity level for the animal.^16^ Although short (0–1 minute in length) and medium (1–10 minutes) bouts of activity were similar across groups (Fig. S2E,F, http://links.lww.com/PR9/A264), longer bouts (greater than 10 minutes) were significantly less likely in CFA- and MIA-injured mice (Fig. 3J). Finally, the Lomb–Scargle periodogram, similar to the χ^2^ periodogram,^48^ allows to evaluate how strong the activity–rest cycle is for different periods (Fig. S2G, http://links.lww.com/PR9/A264). Here, we saw a significant disruption in the 24-hour activity cycle for MIA-injured animals only (Fig. 3K).

Overall, there was not simply a shifting or spreading of activity into light periods, but injured animals, particularly after MIA, were less able to sustain activity during dark periods, requiring more frequent and prolonged rests.

### 3.4. The monoiodoacetate but not the complete Freund adjuvant model induces robust negative affective behaviours

**Figure 4.**
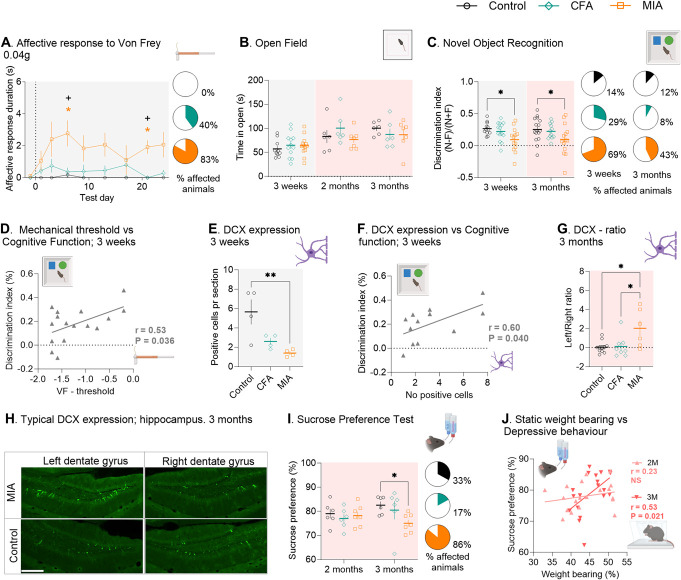
The MIA but not the CFA model induces robust negative affective behaviours. (A) Affective response duration, N = 4-10. (B) Anxiety-like behaviour, assessed using the open field test (N = 6–13). (C) Discrimination index = (nose interaction with novel - familiar) /(nose interaction with novel + familiar). (N = 12–16). (D) Correlation between mechanical threshold and cognitive deficits. (E) DCX expression in the hippocampus assessed using immunohistochemistry (N = 4). (F) Correlation between the number of positive DCX cells and cognitive deficit. (G) DCX expression presented as the ratio between the number of positive cells in the left and right dentate gyrus (N = 11/8/6). (H) Typical expression of DCX in the hippocampus. Scale bar = 100 µm. (I) Depressive-like behaviour assessed by the sucrose preference test (N = 6/6/7). (J) Correlation between static weight-bearing deficit and depressive-like behaviour. Posttest in time-course figures (A); #*P* < 0.05, CFA vs control; *P < 0.05, MIA vs control; +*P* < 0.05 CFA vs MIA, determined using Tukey multiple comparison test. Full analysis outcome in Supplementary Table S1, http://links.lww.com/PR9/A264. For scatter plot figures, posttests between injury groups are displayed using connecting lines *P < 0.05, **P < 0.01, determined using Tukey posttest. Affected animals were defined as responding 1 SD or more from the average performance of the control group. CFA, complete Freund adjuvant; DCX, doublecortin; MIA, monoiodoacetate.

First, we looked at the duration of attentive response to a single application of 3 selected filaments (low = 0.04 g, medium = 0.16 g, high = 1.0 g). This affective response stabilised very rapidly after 10 days and was therefore only measured during the early phase of the pain states to reduce burden on the mice. The MIA model induced stronger affective responses than the CFA model (Fig. 4A, S3A,B,C, http://links.lww.com/PR9/A264), which rarely caused more affective behavior upon stimulation than control. Considering the large variation observed in the affective data collected, we used an approach of individual behavioural profiling, developed to differentiate between “affected” and “exposed but unaffected” animals.^3,49^ Affected animals were defined as responding 1 SD or more from the average performance of the control group. We found that only 40% of CFA-injured animals displayed affective response to the application of filament 0.04 g vs 83% of MIA-injured and 0% for control animals, at 3 weeks post model induction (Fig. 4A).

Unexpectedly, we found little indication of anxiety-like behaviour in either model, using both the open field test and the elevated plus maze, at 3 weeks, 2 months, and 3 months after model induction (Figs. 4B, Fig.S3E, http://links.lww.com/PR9/A264). There was also no sign of differences in crude locomotion between the groups (Fig.S3D, http://links.lww.com/PR9/A264).

However, using the novel object recognition test, we observed significant cognitive deficits, but in the MIA model only, both at 3 weeks and 3 months (Fig.4C, 3 weeks/3 months: MIA: 0.093 ± 0.05/0.097 ± 0.07, CFA: 0.222 ± 0.04/0.223 ± 04, control: 0.266 ± 0.03/0.252 ± 0.05, Fig.S3F,G, http://links.lww.com/PR9/A264). There was an expected decreased recognition of the novel object in the testing phase (Fig. 4C), but also high variability in time exploring each of the 2 identical objects already during the familiarisation, which was unmodified by the introduction of a novel object (Fig. S3F,G, http://links.lww.com/PR9/A264); 29% of CFA-injured animals displayed cognitive deficit vs 69% of MIA-injured animals at 3 weeks (control, 14%), and 8% of CFA-injured animals vs 43% of MIA-injured animals at 3 months (control, 12%). At 3 weeks, there was a correlation between the cognitive function and the mechanical sensitivity (Fig. 4D), and importantly, a significant decrease in DCX-expressing neurons in the hippocampus that are important for learning^54^ in MIA-injured animals (Fig. 4E), which correlated with the cognitive deficit (Fig. 4F). At 3 months, there were no obvious differences in number of DCX-expressing neurons between the groups (Fig. S3H, http://links.lww.com/PR9/A264), but an obvious laterality in DCX expression in the MIA group alone (Fig. 4G,H).

Using the sucrose preference test, we found that only MIA-injured animals developed depressive-like behavior, and not until 3 months after the initiation of the pain states (Fig. 4I, MIA: 75.1 ± 1.4%, CFA: 80.5 ± 3.8%, control: 82.5 ± 1.5% sucrose preference vs water), at which time it correlated with the functional deficit estimated by static weight bearing (Fig. 4J). In total, 17% of CFA-injured animals developed depressive-like behaviour vs 86% of MIA-injured mice (control, 33%).

### 3.5. Exploring novel links between joint inflammation, movement, and pain-related behaviours

**Figure 5.**
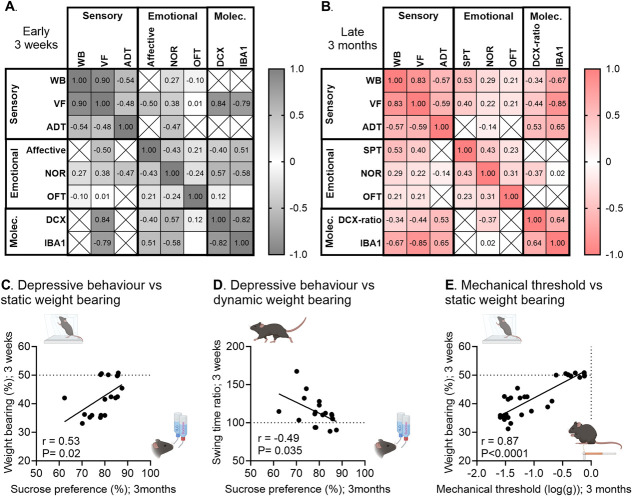
Early weight-bearing deficit in joint disease can be used as a predictor of hypersensitivity and comorbid depressive-like behavior in late disease stage. Summary of correlations between outcome measures recorded on the same animal at the same time point at 3 weeks (A) and 3 months (B) after injury. Values displayed are *r*-values for Pearson *r* correlation analysis, estimating the strength of the correlations. The more intense color coding signifies approaching the perfect fit at −1 or 1. (C–E) Correlations between early weight-bearing deficits at 3 weeks after injury and late outcomes at 3 months after injury. There was a significant correlation between (C) depressive-like behavior at 3 months and static weight-bearing deficit in the first 3 weeks after CFA and MIA injection; (D) depressive-like behavior at 3 months and dynamic weight bearing in the first 3 weeks after CFA and MIA injection; and (E) mechanical threshold at 3 months and static weight-bearing deficit in the first 3 weeks after CFA and MIA injection. See full statistical analysis in supplementary Table S1, http://links.lww.com/PR9/A264, and extended version of the table in Figure S5, http://links.lww.com/PR9/A264. CFA, complete Freund adjuvant; MIA, monoiodoacetate.

We next explored how the different pain-related behaviors and selected molecular outcomes may correlate. Figure 5A, B summarize the correlations between outcome measures recorded for the same animal at 3 weeks (Fig. 5A, S5A, http://links.lww.com/PR9/A264) or 3 months (Fig. 5B, S5B, http://links.lww.com/PR9/A264). As all outcomes were not always measured in all animals, but over different cohorts designed to answer specific questions, correlations could not be performed between all parameters. The sensory and functional outcomes (VF, WB, and ADT) correlated well across models at both the early and the late time points, and changes in these outcome measures were also reflected in the molecular markers, specifically spinal IBA1 and hippocampal DCX. The emotional outcomes, however, did not consistently reflect the sensory and functional outcomes at a given time point. At 3 weeks, the cognitive deficits and affective response to Von Frey application correlated with VF, whereas at 3 months, the depressive and cognitive measures correlated with the WB deficit.

Finally, as predicting the development of significant comorbid affective disorders in patients with joint pain remains a significant clinical challenge, we asked whether early measures of functional deficit, which could be objectively evaluated in the clinics, may be used to predict the late development of comorbid affective disorders and late hypersensitivity. Strikingly, we found that the early measures (average of the first 3 weeks) of both static (WB) and dynamic weight-bearing (swing time) correlated with the depressive-like state as measured at 3 months (Fig. 5C,D). Moreover, there was also a very strong correlation between the early measures of static weight-bearing and the allodynia in the late disease stage (Fig. 5E, full overview of predictive correlations in Table S3, http://links.lww.com/PR9/A264).

To test the relationship between weight-bearing deficits soon after injury (3 weeks) and the likelihood of developing depressive-like behaviour later, we used a generalised linear model with a logit link function. We found that a more balanced weight-bearing immediately at 3 weeks is related to a lower likelihood to develop depressive-like behaviour at 3 months and that an animal with a weight-bearing of 41.5% has 50% chance of developing depressive-like behaviour (Fig. S4, http://links.lww.com/PR9/A264). This implied that early weight-bearing deficit, a correlate for the pain associated with limb-use, could indeed be used to estimate the development of comorbid depressive-like behaviour at 3 months.

## 4. Discussion

Here, we show that different models of joint pain may lead to sensory outcomes with subtle differences, but strikingly distinct functional, affective, and molecular outcomes. Moreover, we found that the early changes in gait/weight bearing correlated with the likelihood of developing affective comorbidities such as depressive-like symptoms in the long term.

Only the MIA model showed significant affective comorbidities, such as cognitive deficits, increased affective response to mechanical stimuli, as well as depressive-like behaviour. Other studies have previously detected depressive-like behaviour and cognitive deficits in rodent models of persistent pain, including the MIA model, although timelines and occurrence of comorbid emotional changes in preclinical pain models are very variable and at times contradicting.^1,40,56^ In our study, depressive-like behaviors were only assessed in the chronic stages, as other preclinical studies suggested that depressive-like behavior requires at least 4 to 8 weeks to develop robustly.^50,56^ Indeed, no depressive-like behavior was detected at 2 months, whereas anxiety-like behavior was never detected. The absence of anxiety-like behavior aligns with similar studies^46^ but not with others,^41,42^ reflecting the well-known complexity of the field.^15^ Disparities may be related to factors like strain,^27^ age of the test-subjects,^34^ experimental design-choices modulating stress,^43^ behavioural assays^15^ or injury-related parameters.^47^ It is therefore possible that modifying any of these parameters could change our observations.

It is important to note that not all MIA-injured animals developed negative affective outcomes. Indeed, although we observed very little variation in the sensory and functional outcome measures within each injury group, the variation was much greater for affective behaviours. Therefore, we also focused on individual animal behavior, as others have done before.^3,49^ With this approach, we found that around 86% of MIA-injured animals were presenting with depressive-like behavior and 57% with cognitive deficits, 3 months after disease onset. Similarly, only about 17% of patients with rheumatoid arthritis have major depressive disorder. However, depression in patients with arthritis is crucially associated with poor long-term outcomes, increased pain, fatigue, and physical disability.^30^ It is therefore paramount to improve the understanding of mechanisms underlying chronic pain in those patients with affective comorbidities and future studies comparing animals that develop comorbid depression with nonaffected animals could unveil new potential therapeutic targets for this subset of patients.

The MIA and CFA models also showed significant differences in gait, most prominently in the early stages of the joint pain states. The MIA injury produced compensatory mechanisms as seen by increased duration of the swing and length of the stride of the injured leg, and other output measures indicating a reduction of the load on the injured leg. Interestingly, it is also in the early days after MIA injection that mice displayed the strongest affective response to mechanical stimuli and disruption of activity and sleeping patterns, indicating that the early stage of the MIA model was the most unpleasant to the animals. Surprisingly, the measure of activity using the Open Field test did not show any differences in locomotion after injury, showing the importance of measuring mice activity in their home cage across days rather than a short period under experimental conditions. Similar misalignment can be seen between results from the static weight bearing vs the Catwalk gait analysis. Static weight-bearing measures, which require the animals to sit still for a while and distribute the bodyweight across the 2 hind legs, suggested clear and prolonged weight-bearing deficits in both models. However, in the Catwalk, animals walk along a corridor in a goal-oriented manner, using all 4 limbs, which lead to less signs of deficits at later stages, as reported by others.^23,24^ Crucially, in the chronic phase of the disease, we found a significant correlation between static weight-bearing deficits and mechanical sensitivity, suggesting that both measures may be equally useful as a measure of pain-related outcome. Overall, the CFA and MIA models lead to different compensatory gait adaptation, with milder deficits from the CFA injury, which may reflect either compound-related effects, volume/dose choices, or differences between an ankle and a knee injury.^2^ Weight-bearing asymmetry and gait changes are thought to reflect pain related to limb use, and together with the other enhanced pain-related outcomes in the MIA-model, our results suggest an overall greater pain burden in this model, which may be driving the long-term comorbidities.

One significant limitation of our work was the fast onset of the models because human joint pain does not develop in such short time frame.^29^ Our study was also performed in a restricted group of mice (8-week-old males at the onset of the disease, with similar weight and identical diet), although many factors are known to influence the outcome of joint diseases, including age, sex, and body mass index.^35^ Sex differences in joint diseases have not often been studied, even less so in the context of interactions with affective comorbidities. As females are known to be more sensitive to chronic pain and stress-related conditions,^12,21,22^ , and have different circadian and activity pattern,^31,32^ it is important for our findings to be validated in females. Very few studies have investigated outcomes across different joint pain models to differentiate model-specific behavioural and molecular outcomes versus those reflecting generalised chronic pain pathobiology.^20,57^ Here, for the first time, we have identified correlations between early functional outcomes and late affective and sensory measures. Our results suggest that the intense sensory, functional and affective changes in the early stages of the disease may be driving the long-term comorbidities. Therefore, we propose that early functional deficit, reflecting the overall pain burden, may be a useful predictor of late comorbid depressive-like state and hypersensitivity, and that potential early intervention may prevent the later development of comorbid depression. Future experiments should explore if similar early model-specific measures have predictive value in other models of persistent pain.

## Disclosures

The authors have no conflict of interest to declare.

## Supplemental digital content

Supplemental digital content associated with this article can be found online at http://links.lww.com/PR9/A264.
