# Supplementary material for: Differences in multidimensional phenotype of 2 joint pain models link early weight-bearing deficit to late depressive-like behavior in male mice
Source: Pain Rep. 2024 Nov 20;9(6):e1213. doi: 10.1097/PR9.0000000000001213 (PMC11581759; doi:10.1097/PR9.0000000000001213)
Supplement: Supplementary file 1 [file painreports-9-e1213-s001.pdf]

**Differences in multidimensional phenotype of two joint pain models link early weight bearing deficit to late depressive-like behavior in male mice**

Sara Hestehave[1,2], Roxana Florea[1], Alexander J.H. Fedorec[1], Maria Jevic[1], Lucile Mercy[1], Annia Wright[1], Oakley B. Morgan[1], Laurence A. Brown[3], Stuart N. Peirson[4], Sandrine M. Géranton[1*].

## Supplementary Data

## Methods and materials

### *Animals and housing;*

For all experiments, male mice (C57Bl/6J from Charles River, UK) arrived at our facility at 8 weeks of age, and were left to acclimatize for at least 7 days before experiments started. All animals were kept in groups of 4-5 in a temperature-controlled (20±1°C) environment in Individual Ventilated Cages-cages (SealSafe Plus GM500, Tecniplast, 39*19*16cm) equipped with sawdust (Lignocel Select fine), nesting material (Datesand Cocoons), wooden chew stick (LBS Small Aspen chewstick) and cardboard tunnels for shelter and handling (LBS Standard Fun tunnel); light-dark cycle of 12 hours (gradual lights on between 7-8a.m. and off between 7-8p.m); and ad libitum provision of food and water (Teklad global 2018 diet). The only exception to these housing-conditions was during the sleep/activity-recordings, where animals were single-housed in open wire top M1 mouse cage (North Kent Plastic Cages, 45*28*13cm) without tunnels for shelter. All experiments were carried out under the Home Office License P8F6ECC28, and all efforts were made to minimize animal suffering and to reduce the number of animals used (UK Animal Act, 1986).

### *Study design;*

This study focused on male mice, as its aim was to explore differences between pain models in terms of sensory, functional and emotional outcomes. We therefore decided to strengthen our statistical power towards treatment outcome (i.e. CFA *vs* MIA) which necessitate high n numbers when looking at emotional parameters, due to expected high variability in these behavioral measures. No specific power-calculation was performed for these studies, but group-sizes required for each type of behavioral outcome were estimated based on previous experience with the specific outcome measures in our group. Moreover, the oestrus cycle is known to affect locomotor activity patterns in females [13,14], which would have meant much longer monitoring and larger sample sizes needed for our activity and sleeping patterns studies.

The study was divided in cohorts/studies including animals from all experimental groups (CFA vs MIA vs control, n = 4-8, depending on the study). Across all cohorts, a total of 127 animals were used in these experiments. Individual animals were considered as experimental units, and animals were allocated to injury-group using block randomization and computer generated random allocation sequence, to secure that animals from each group were represented in each home cage in order to minimize the risk of potential confounders affecting the groups unequally.

In order to not stress the mice from over-testing, and thereby confounding the results, each experiment was designed to test only a selection of parameters. All injury-groups and animals in a given cohort were exposed to the same procedures and assessments. Experiments were terminated at different time points in order to collect tissue at the appropriate time point for various molecular markers. Therefore, group-sizes were variable throughout the study, but always distributed equally across treatment. Group-sizes are presented as a range throughout the manuscript. As the models used in these experiments often produce significant visual characteristics like joint-swelling and decreased use of the affected limb, that are obvious visually when handling and assessing behavioral outcomes, it was not possible to blind the experimenter to injury-group for outcomes involving manual observations, like mechanical and cold allodynia. When possible, automatic objective measures were used for behavioral assessment using Ethovision, Catwalk gait analysis, sleep/activity tracking or static weight-bearing. For immunohistochemistry-analysis, the experimenters were always blinded to injury-group while counting/analysing results.

***Experimental procedures;***

*Induction of injury, (CFA);* Induction of the Complete Freund's Adjuvant model of tibio-tarsal joint inflammation (CFA) was performed similar to what was previously reported by our group [17]. Animals were anaesthetized in an induction chamber using Isoflurane 2.5% mixed in $O_2$ at a flowrate of 1.5 L/min, and maintained via facemask at 2.0% during the injection. Anaesthetic depth was confirmed by lack of withdrawal reflex to a pinch to the tail. The animal was then placed in lateral recumbency on the right side, for fixation of the left ankle joint. Using a precision Hamilton-syringe, a 25G needle entered the ankle joint from the lateral posterior position, with the ankle kept in plantar flexion to open the joint, and 5µl of CFA (Complete Freund's Adjuvant, Sigma) was injected.

*Induction of injury, (MIA);* Induction of the Monoiodoacetate Arthritis Model to the knee joint (MIA) was performed similar to previously reported [21]. On the day of injection, the monoiodoacetate solution was freshly prepared in sterile saline for injection of 1mg Sodium Iodoacetate (≥98%, Sigma, I2512-25G,) in 10µl saline (9% NaCl). Anesthesia was induced similarly to above, but the animal was placed on the back in dorsal recumbency, and the fur was shaved in the area around the knee on the left hindleg. The knee was stabilized and fixed in a slightly bend position and the patellar tendon was visualized as a white line below the skin. The injection of 10µl using a 30G insulin-syringe (BD Micro-Fine Plus Demi, 0.3ml (30G) 8mm) was made intraarticularly in the joint-space by applying it perpendicularly through the tendon just below the patella, and with as minimal movement as possible. Note that the compound is very toxic, and that even small volumes delivered subcutaneously outside of the joint-space, or potential damage to the popliteal artery in the knee, may cause intoxication of the animal. Great care must be taken to secure a controlled injection and to secure accurate volume to not risk leakage of the fluid from the joint space.

*Control animals.* Control animals were only exposed to anesthesia.

***Behavioural testing;***

Behavioural testing was always performed in randomized order and by the same female experimenter. Animals were always allowed at least 30min of habituation to the testing room prior to behavioural testing. Unless otherwise specified behavioral tests were always performed between 8am and 2pm.

*Mechanical allodynia (VF);* Ipsilateral (left) paws were tested throughout the study. Low intensity mechanical sensitivity assessment was similar to previously reported by our group [18], using a series of calibrated von Frey monofilaments (0.02; 0.04; 0.07; 0.16; 0.4; 0.6; 1.0) (Ugo Basile SRL, Italy). Animals were placed in Plexiglas chambers, located on an elevated wire grid, and allowed to habituate to the testing environment for at least 60min prior testing. Once the animals were calm, the plantar surface of the paw was stimulated, starting with a 0.6g filament applied with uniform pressure for 5seconds. A brisk withdrawal, stretching or licking of toes, was considered as a positive response, whereupon the next lower-force filament was applied. In the absence of a positive response, the next higher-force filament was applied in the next test. After the first change in response-pattern, suggesting the threshold, additional 4 stimulations were applied; applying the next higher-force filament when no response, and lower-force filament following positive responses. The response pattern determined the constant, *k* [9], and the 50% response threshold was determined using the following equation; 50% threshold (g) = $10^{\log(\text{last filament})+k*0.3}$.

*Affective-motivational behaviour (AF);* In addition to assessing pure reflexive sensory threshold to mechanical stimulation (VF), we also adopted and modified the protocol described by Corder et al. and Maiarù et al. [8,16] to assess the affective responses displayed after stimulation with three selected filaments (low; 0.04g, medium; 0.16g, high; 1.0g). The assessment was always performed following the VF-assessment, while the animals were still in the Plexiglas chamber, and following a period of rest. Starting with the low-intensity filament, each filament was applied once for 1sec, and the duration of affective response was recorded as the amount of time the animal showed conscious attending behavior to the stimulated paw by licking, biting, lifting, looking at or guarding the paw, within 30 seconds after application of the filament. The animal was left to rest for at least 10min before the next higher filament force was applied.

*Cold allodynia, (ADT):* While the animals were still in the plexiglas chambers following von Frey measurements, cold allodynia was assessed using application of a drop of acetone (Acetone Drop Test, ADT) to the plantar surface of the paw, using a plastic syringe without mechanically touching the skin. Following application, the duration of the response was then recorded, with a maximum of 30 seconds. A positive response was considered as flinching, licking, looking at or withdrawing the paw. The application and assessment was performed 2-3 times on each paw for each animal with 5-10 minutes between each application, and the average of the measurements was calculated.

*Functional impairment - Weight bearing (WB);* To assess the functional impact of the injury the static weight bearing distribution was assessed similarly to previously described [15]. Hindlimb weight bearing was measured using a Bioseb Incapacitance Test (Bioseb) which measures the weight distribution across the two hindlimbs of a stationary animal. Animals were habituated and trained to become comfortable with the testing paradigm during short sessions for 5 days prior to induction of the model. Three readings were collected and averaged for each animal, and the

weight borne by the ipsilateral limb was expressed as a percentage of the weight borne across both hindlimbs (WB-% = (weight borne on the injured leg/weight borne on both legs) * 100%).

*Catwalk gait analysis:* Analysis of voluntary movement and gait pattern was performed using the Catwalk® XT 10.0 system (Noldus Information Technology) [23], and based on our previous experience [11]. For optimal contrast for the recording, testing was conducted in a dark room. Briefly, green light was internally reflected into a glass plate, on which an enclosed corridor was fixed, with red backlight above the corridor. A video-camera was mounted underneath the setup and recorded the paw prints being lit up by the green light when paws were in contact with the glass plate as the animal walked along the corridor. A run was regarded as compliant when the animal entered in one end of the corridor and moved fluently across the plate towards the other end of the corridor, with a running duration below 12 seconds and a maximum variation below 75%. Three compliant runs were recorded for each animal, with no previous training/habituation or food-deprivation. Following the recording, compliant runs were classified and cleaned/corrected for potential miscellaneous prints. For outcome-measures like swing-time ratio, contact area and single stance, the data was converted into a ratio between ipsi- and contra-lateral hind-limbs. The term "print position" refers to the distance between the position of the hindpaw, and the previously placed front paw. No prior habituation to the equipment or training was needed for the animals to complete the desired compliant runs.

*Sucrose Preference Test (SPT);* In order to assess depressive-like behaviour, the sucrose preference test was included as a measure of anhedonia [24], At least 5 days before SPT each home-cage was fitted with two water bottles, in order to have the mice accustomed to drinking from both bottles / sides of the cage. Two days before the SPT test, one of the water-bottles was filled with 1% sucrose solution for approximately 24h, with the two bottles changing side half way, to allow the mice time to discover the sweet solution, and learn that it may be presented in both sides. No sucrose was provided the last 24 hours before the actual test. For the SPT test, all animals were individually housed in clean cages similar to their home-cage environment for 12h overnight (7pm-7am), provided similar enrichment, nesting material and food on the same IVC-rack as their home-cage, and were given free access to two pre-weighed bottles containing normal drinking water or 1% sucrose solution. In the morning all bottles were weighed and mice were placed back together with their cage-mates. This re-introduction released some fighting in some of the cages, and mice were given the following night to calm down before the test was repeated overnight with the sucrose bottle presented in the opposite side, to account for potential side-preference, as reported previously in rats [11]. No side-preference was detected in the current study, and the sucrose preference % was calculated on the total amount consumed across the two nights combined, using the following calculation; SPT% = (sucrose-solution consumed / (total fluid consumption)) * 100%. The sucrose-solution was always prepared fresh before provision at 1% in the normal drinking water. As recent meta-analysis suggests food- and water-deprivation to confound the outcome of this assay [4]; no water- or food-deprivation was employed in this study.

*Elevated Plus Maze (EPM);* To assess anxiety-like behaviour an EPM was used as described previously [11,12], but for these experiments adopted to mice rather than rats. The maze consisted of four arms (35*5 cm) arranged in a cross-like disposition, and 60cm above ground

(Ugo Basile SRL, Italy). Two opposite arms were open, and the other two were equipped with 15 cm high walls on each side for enclosement. All were connected by a central 5*5 cm square. The animal was placed in the centre, for free exploration for 5 minutes. Recording was performed by use of a camera placed above the maze, and movement between zones was tracked using EthoVision XT14 (Noldus Information Technology).

*Open Field Test (OFT);* To assess anxiety-like and locomotor activity, an OFT was used, using similar approach to previously described [10]. The OFT was a circular open arena with grey plastic flooring and blue plastic sides (diameter, 36cm, height 32cm). The arena was evenly illuminated by lighting placed above the arena. A video camera was positioned directly above the arena and connected to a computer performing live-tracking and recording of the behavior using Ethovision XT14. The animal was placed in the middle of the arena and allowed 5 minutes of freely exploration. The proportion of time spent in the centre vs by the edges/walls of the arena, was used as a marker for the anxiety-like behavior (outer zone; 6.7cm along the edge of the arena. Centre zone diameter; 22.6cm).

*Novel Object Recognition (NOR):* The Novel Object Recognition test was performed similarly to previously described [5,10,20], with some modifications. Testing was carried out in the same circular arena as the OFT, and always on the following day, making the OFT also serve as habituation to the arena without objects. A video camera was positioned directly above the arena and connected to a computer performing live-tracking and recording of the behavior using Ethovision XT14, with 3-point tracking of nose-center-tail points. The familiar/similar objects were brown circular glass bottles (d; 7cm, h; 18cm), while the "novel" object was a translucent elliptical glass bottle (d; 9*5cm, h; 15cm) containing white sand for coloring. The animal was placed in the arena facing away from the objects, and first allowed 10minutes freely exploration and habituation to the arena including the two similar brown glass bottles (making these "familiar object" following the habituation phase), placed in two opposite quadrants of the arena. Following habituation, the animal was rejoined with its cage-mates. Three hours later, the animal was reintroduced to the arena for a 5min test, where one of the familiar objects had been replaced with the novel object, displaying a different color and shape, but same texture and sensation upon manipulating. The object replaced / side was alternated between test subjects and experimental groups to randomize for potential side-preferences. Upon completion, all recordings/trackings were corrected for appropriate nose/tail-tracking by the software, and exploration of an object was defined as the nose being within approximately 2cm of the objects. The proportion of time spent exploring the objects was assessed by calculating a percentage of time exploring the novel object, or for the habituation-phase; the object which was later replaced by the novel object. Using the following formula;

$$Discrimination\ index = \frac{\text{Time spent exploring novel} - \text{familiar object}}{\text{Time spent exploring novel} + \text{familiar onject}}$$

*Joint circumference (JC)*; Joint circumference was assessed to give an indication of the inflammatory development after the two injuries. It was measured on all left ankle and knee-joints in one cohort of animals, while the animal was already anaesthetized immediately before induction of the injury, and immediately before perfusion of the individual animal. The lateromedial (LM) and dorsoplantar (DP) diameters were measured using calipers, and the

circumference was calculated using an approximation of the perimeter of an ellipse; $C = 2 \times \pi \times \sqrt{0{,}5 \times (a^2 + b^2)}$, where a = the radius of DP and b = the radius of LM [2,3].

*Sleep/activity-pattern;* To measure undisturbed activity in the home-cages, we adopted the approach of Brown et al. [7] using non-invasive passive infrared motion sensors. Animals were single-housed with a 12h light:12h dark cycle. The cages, were open with wire tops (M1 mouse cage, North Kent Plastic Cages, floor area: 500cm$^2$) and a passive infrared (PIR) motion sensor was fitted above the cage. For accurate measurements of activity, the area below the food- and water-hopper was blocked off and tunnels / shelters were removed, allowing the animal to be in the sensors receptive field at all times. For these experiments, due to restriction on the time animals could remain single housed, animals were first injected and then placed directly into the recording cages, where they remained for a week of uninterrupted recording. Home cage mouse activity was tracked as in Brown et al. [7], with measurements taken every 10 seconds across multi-day periods. As before, sleep was defined as periods in which no activity was measured for 40 seconds or more. Activity and sleep data were smoothed by calculating the mean in 10 minute bins as preliminary experiments demonstrated that this provided a balance between reducing measurement noise and maintaining time series features. Several summary statistics of circadian disruption were calculated for individual animals across the 7 day period and, where appropriate, on each individual day: inter-daily stability, intra-daily variability, light-phase activity, dark-phase sleep, the Lomb-Scargle periodogram, similar to the chi-square periodogram [6].

*Immunohistochemistry;* For immunohistochemistry, mice were deeply anaesthetized with pentobarbital, weighed (BW), and perfused transcardially, first with heparinized saline (5000IU/mL), followed by freshly made 4% paraformaldehyde in 0.1M phosphate buffer (20mL pr adult mouse). Spinal cord and brain were dissected out and postfixed in the same paraformaldehyde solution for 2 hours, before being transferred to 30% sucrose solution in PB with 0.01% $NaN_3$ at 4°C until cutting, at least 3 days after perfusion. The tissue was cut on a freezing microtome at 40µm thickness.

Lumbar spinal cord and hippocampal sections were rinsed in 0.1M PB and subsequently incubated at room temperature for 1h with 30% (v/v) normal goat serum (Invitrogen, Cat#31872) in 0.1M PB containing 10% Triton X-100. Sections were then incubated overnight at RT with one of the following primary antibodies: guinea pig anti-c-Fos (1:2000, Synaptic Systems, Cat#226004), rabbit anti-CGRP (1:5000, Millipore, AB5920), rabbit anti-IBA1 (1:500, Synaptic Systems, Cat#234008), rabbit anti-GFAP (1:4000, Dako, Z0334) or goat anti-DCX (1:500, Santa Cruz Biotechnology, F2916). Next day sections were washed three times in 0.1M PB and then incubated in darkness for 2h at RT with the respective secondary antibodies diluted in TTBS at 1:500. Lastly, after final washes, sections were mounted on gelatinized slides, coverslipped with Fluoromount Aqueous Mounting Medium (Sigma-Aldrich, F4680), and stored at 4ºC.

*Microscopy and quantification of immunofluorescence*

Cell counting of c-Fos positive neurons (spinal cord sections from Lumbar L4 to L6) was performed directly under the microscope. For the other stains, images were collected using a Leica (Nussloch, Germany) DMR microscope connected to a Hamamatsu (C4742-95; Shizuoka, Japan) digital CCD camera and Volocity 6.3 Software. Intensity of CGRP immunoreactivity in superficial

laminae LI-II as well as GFAP stain were analyzed using the 'Mean grey value' plugins in ImageJ. Background intensity of Laminae IV-V was subtracted from the positive signal in LI-II. Finally, IBA1 positive microglia in laminae I to III of the lumbar cord L4 to L6 and DCX positive cells in the dentate gyrus were manually counted from pictures using the counter feature in ImageJ. In all cases, 6 sections per mouse were analysed. Spinal cord sections had a cut in the contralateral ventral horn and the brain sections had a cut in the left through the hypothalamus for orientation purposes.

*Data and statistical analysis;* All statistical tests were performed in IBM SPSS Statistic Program (vers. 26) or GraphPad Prism (vers 9), and P<0.05 was considered statistically significant. Repeated Measures ANOVA was used when appropriate, but when some experiments/cohorts were terminated early due to the Covid-19 pandemic or tissue-collection, the data-sets affected were analyzed using Mixed-effects model to accommodate the missing data for the remaining period, while still including what had been obtained. Pearson r was used for correlation analysis. All details on statistical analysis, factors tested and significant outcomes, can be found in supplementary tables S1 and S2.

Weighted average was calculated as an Area Under the Curve for each individual animal tested, and the value was divided by the number of days in the experimental period, thereby giving a meaningful value to compare the overall differences between groups. For the majority of outcome measures, where we explored long term outcomes, we calculated a weighted average for two different periods defining the phases of the injury, "early" and "late". The phases were defined as Early; from induction to around day 22, Late; from day 23 onwards (or the first data-collection/test following the day 23 test). The VF-data-set was log-transformed to ensure a normal distribution, as the von Frey hairs are distributed on an exponential scale. This was similar to previous studies in our group [18], and others [19].

We also used an approach of individual behavioural profiling, developed to differentiate between "affected" and "exposed but unaffected" animals [1,22]. Affected animals were defined as responding one standard deviation or more from the average performance of the control group. This calculation allowed to assess the percentage of affected animals within each treatment group.

All animals that completed the experiments, were included in the analysis, and no exclusion-criteria were set beforehand for inclusion in experiments or data-analysis.

## References

[1] Ardi Z, Albrecht A, Richter-Levin A, Saha R, Richter-Levin G. Behavioral profiling as a translational approach in an animal model of posttraumatic stress disorder. Neurobiol Dis 2016;88:139–147.

[2] Berke MS, Colding-Jørgensen P, Pedersen LG, Hestehave S, Kalliokoski O, Jensen HE, Sørensen DB, Hau J, Abelson KS. Effects of Transdermal Fentanyl Treatment on Acute Pain and Inflammation in Rats with Adjuvant-induced Monoarthritis. comp med 2022;72:320–329.

[3] Berke MS, Fensholdt LKD, Hestehave S, Kalliokoski O, Abelson KSP. Effects of buprenorphine on model development in an adjuvant-induced monoarthritis rat model. PLoS ONE 2022;17:e0260356.

[4] Berrio JP, Hestehave S, Kalliokoski O. Reliability of sucrose preference testing following short or no food and water deprivation—a Systematic Review and Meta-Analysis of rat models of chronic unpredictable stress. Transl Psychiatry 2024;14:39.

[5] Bevins RA, Besheer J. Object recognition in rats and mice: a one-trial non-matching-to-sample learning task to study "recognition memory." Nature Protocols 2006;1:1306–1311.

[6] Brown L, Fisk A, Pothecary C, Peirson S. Telling the Time with a Broken Clock: Quantifying Circadian Disruption in Animal Models. Biology 2019;8:18.

[7] Brown LA, Hasan S, Foster RG, Peirson SN. COMPASS: Continuous Open Mouse Phenotyping of Activity and Sleep Status. Wellcome Open Res 2016;1:2.

[8] Corder G, Tawfik VL, Wang D, Sypek EI, Low SA, Dickinson JR, Sotoudeh C, Clark JD, Barres BA, Bohlen CJ, Scherrer G. Loss of μ opioid receptor signaling in nociceptors, but not microglia, abrogates morphine tolerance without disrupting analgesia. Nat Med 2017;23:164–173.

[9] Dixon WJ. Efficient Analysis of Experimental Observations. Annu Rev Pharmacol Toxicol 1980;20:441–462.

[10] Gomez K, Stratton HJ, Duran P, Loya S, Tang C, Calderon-Rivera A, François-Moutal L, Khanna M, Madura CL, Luo S, McKiver B, Choi E, Ran D, Boinon L, Perez-Miller S, Damaj MI, Moutal A, Khanna R. Identification and targeting of a unique $Na_V$ 1.7 domain driving chronic pain. Proc Natl Acad Sci USA 2023;120:e2217800120.

[11] Hestehave S, Abelson KSP, Brønnum Pedersen T, Finn DP, Andersson DR, Munro G. The influence of rat strain on the development of neuropathic pain and comorbid anxio-depressive behaviour after nerve injury. Sci Rep 2020;10:20981.

[12] Hestehave S, Abelson KSp, Brønnum Pedersen T, Munro G. Stress sensitivity and cutaneous sensory thresholds before and after neuropathic injury in various inbred and outbred rat strains. Behavioural Brain Research 2019;375:112149.

[13] Krizo JA, Mintz EM. Sex differences in behavioral circadian rhythms in laboratory rodents. Front Endocrinol (Lausanne) 2014;5:234.

[14] Kuljis DA, Loh DH, Truong D, Vosko AM, Ong ML, McClusky R, Arnold AP, Colwell CS. Gonadal- and sex-chromosome-dependent sex differences in the circadian system. Endocrinology 2013;154:1501–1512.

[15] Learoyd AE, Sen D, Fitzgerald M. The pain trajectory of juvenile idiopathic arthritis (JIA): translating from adolescent patient report to behavioural sensitivity in a juvenile animal model. Pediatr Rheumatol 2019;17:60.

[16] Maiarù M, Leese C, Davletov B, Hunt SP. The alleviation of neuropathic pain behaviours by a single injection of a synthetic substance P-botulinum conjugate persists for up to 120d and can be restored with a second injection of conjugate. Neuroscience, 2020 doi:10.1101/2020.12.28.423185.

[17] Maiarù M, Morgan OB, Mao T, Breitsamer M, Bamber H, Pöhlmann M, Schmidt MV, Winter G, Hausch F, Géranton SM. The stress regulator FKBP51: a novel and promising druggable target for the treatment of persistent pain states across sexes. Pain 2018;159:1224–1234.

[18] Maiarù M, Tochiki KK, Cox MB, Annan LV, Bell CG, Feng X, Hausch F, Géranton SM. The stress regulator FKBP51 drives chronic pain by modulating spinal glucocorticoid signaling. Sci Transl Med 2016;8:325ra19.

[19] Mills C, LeBlond D, Joshi S, Zhu C, Hsieh G, Jacobson P, Meyer M, Decker M. Estimating Efficacy and Drug ED50's Using von Frey Thresholds: Impact of Weber's Law and Log Transformation. The Journal of Pain 2012;13:519–523.

[20] Moriarty O, Gorman CL, McGowan F, Ford GK, Roche M, Thompson K, Dockery P, McGuire BE, Finn DP. Impaired recognition memory and cognitive flexibility in the ratL5–L6 spinal nerve ligation model of neuropathic pain. Scandinavian Journal of Pain 2016;10:61–73.

[21] Pitcher T, Sousa-Valente J, Malcangio M. The Monoiodoacetate Model of Osteoarthritis Pain in the Mouse. Journal of Visualized Experiments 2016. doi:10.3791/53746.

[22] Sarkar I, Snippe-Strauss M, Tenenhaus Zamir A, Benhos A, Richter-Levin G. Individual behavioral profiling as a translational approach to assess treatment efficacy in an animal model of post-traumatic stress disorder. Front Neurosci 2022;16:1071482.

[23] Vrinten DH, Hamers FFT. 'CatWalk' automated quantitative gait analysis as a novel method to assess mechanical allodynia in the rat; a comparison with von Frey testing. Pain 2003;102:203–209.

[24] Willner P, Towell A, Sampson D, Sophokleous S, Muscat R. Reduction of sucrose preference by chronic unpredictable mild stress, and its restoration by a tricyclic antidepressant. Psychopharmacology 1987;93. doi:10.1007/BF00187257.

**Supplementary data**

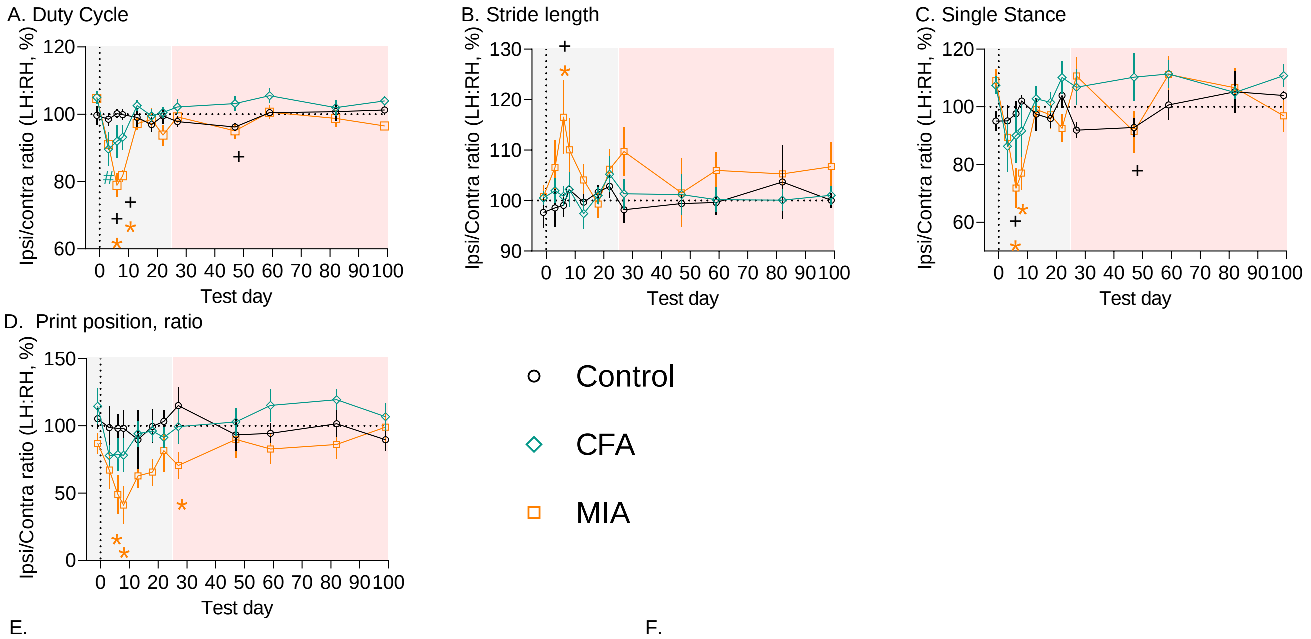


E.

| Peak Day 8 | Static WB (WB) | Single stance (Catwalk) | Stride length (Catwalk) | Duty cycle (Catwalk) | Print position (Catwalk) | Swing time (Catwalk) |
|---|---|---|---|---|---|---|
| WB: Static WB | 1.00 | 0.62 | -0.36 | 0.85 | 0.64 | -0.77 |
| Catwalk: Single stance | 0.62 | 1.00 | 0.22 | 0.81 | 0.34 | -0.60 |
| Catwalk: Stride length | -0.36 | 0.22 | 1.00 | -0.32 | -0.57 | 0.62 |
| Catwalk: Duty cycle | 0.85 | 0.81 | -0.32 | 1.00 | 0.73 | -0.86 |
| Catwalk: Print position | 0.64 | 0.34 | -0.57 | 0.73 | 1.00 | -0.68 |
| Catwalk: Swing time | -0.77 | -0.60 | 0.62 | -0.86 | -0.68 | 1.00 |

Scale: 1.0, 0.5, 0, -0.5, -1.0

F.

| Early 3 weeks | Static WB (WB) | Single stance (Catwalk) | Stride length (Catwalk) | Duty cycle (Catwalk) | Print position (Catwalk) | Swing time (Catwalk) |
|---|---|---|---|---|---|---|
| WB: Static WB | 1.00 | 0.35 | -0.23 | 0.40 | 0.39 | -0.55 |
| Catwalk: Single stance | 0.35 | 1.00 | 0.14 | 0.72 | 0.14 | -0.43 |
| Catwalk: Stride length | -0.23 | 0.14 | 1.00 | -0.53 | -0.72 | 0.69 |
| Catwalk: Duty cycle | 0.40 | 0.72 | -0.53 | 1.00 | 0.68 | -0.81 |
| Catwalk: Print position | 0.39 | 0.14 | -0.72 | 0.68 | 1.00 | -0.70 |
| Catwalk: Swing time | -0.55 | -0.43 | 0.69 | -0.81 | -0.70 | 1.00 |

Scale: 1.0, 0.5, 0, -0.5, -1.0

G.

| Late 3 months | Static WB (WB) | Single stance (Catwalk) | Stride length (Catwalk) | Duty cycle (Catwalk) | Print position (Catwalk) | Swing time (Catwalk) |
|---|---|---|---|---|---|---|
| WB: Static WB | 1.00 | 0.34 | -0.28 | 0.52 | -0.20 | -0.47 |
| Catwalk: Single stance | 0.34 | 1.00 | 0.46 | 0.84 | -0.44 | -0.14 |
| Catwalk: Stride length | -0.28 | 0.46 | 1.00 | 0.04 | -0.54 | 0.79 |
| Catwalk: Duty cycle | 0.52 | 0.84 | 0.04 | 1.00 | -0.15 | -0.53 |
| Catwalk: Print position | -0.20 | -0.44 | -0.54 | -0.15 | 1.00 | -0.35 |
| Catwalk: Swing time | -0.47 | -0.14 | 0.79 | -0.53 | -0.35 | 1.00 |

Scale: 1.0, 0.5, 0, -0.5, -1.0

H.. Body weight gain

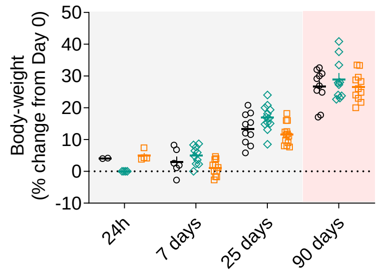

**Figure S1: MIA, but not CFA, induces prominent changes in dynamic gait, specifically in the early stages of the disease state**. **(A)** Duty Cycle, **(B)** Stride length, **(C)** Single stance and **(D)** Print Position were all affected by MIA injected in the knee joint. **(E-G)** Correlations between static weight bearing and catwalk outcome measures recorded in the same animal on the same day at Peak-Day 8, Early – 3 weeks and Late – 3months after injury. The r-values displayed signify the strength of the fit, as determined using Pearson r correlation analysis, and the higher intensity of the colors, the closer to the perfect fit at 1 or –1. **(H)** Body weights were monitored throughout the experiment and were no different from control animals. (**A-D**) Data shows mean ± S.E.M. Post-test in time-course figures (**A, B, C, D, H**); $^{\#}P<0.05$, CFA *vs* control; $^{*}P<0.05$, MIA *vs* control, $^{+}P<0.05$ CFA *vs* MIA, as determined using Tukey's multiple comparison test. Full analysis-outcome in Supplementary **Table S2**. N =6/6/7 for **A-D**. N=2-13 for **F**.

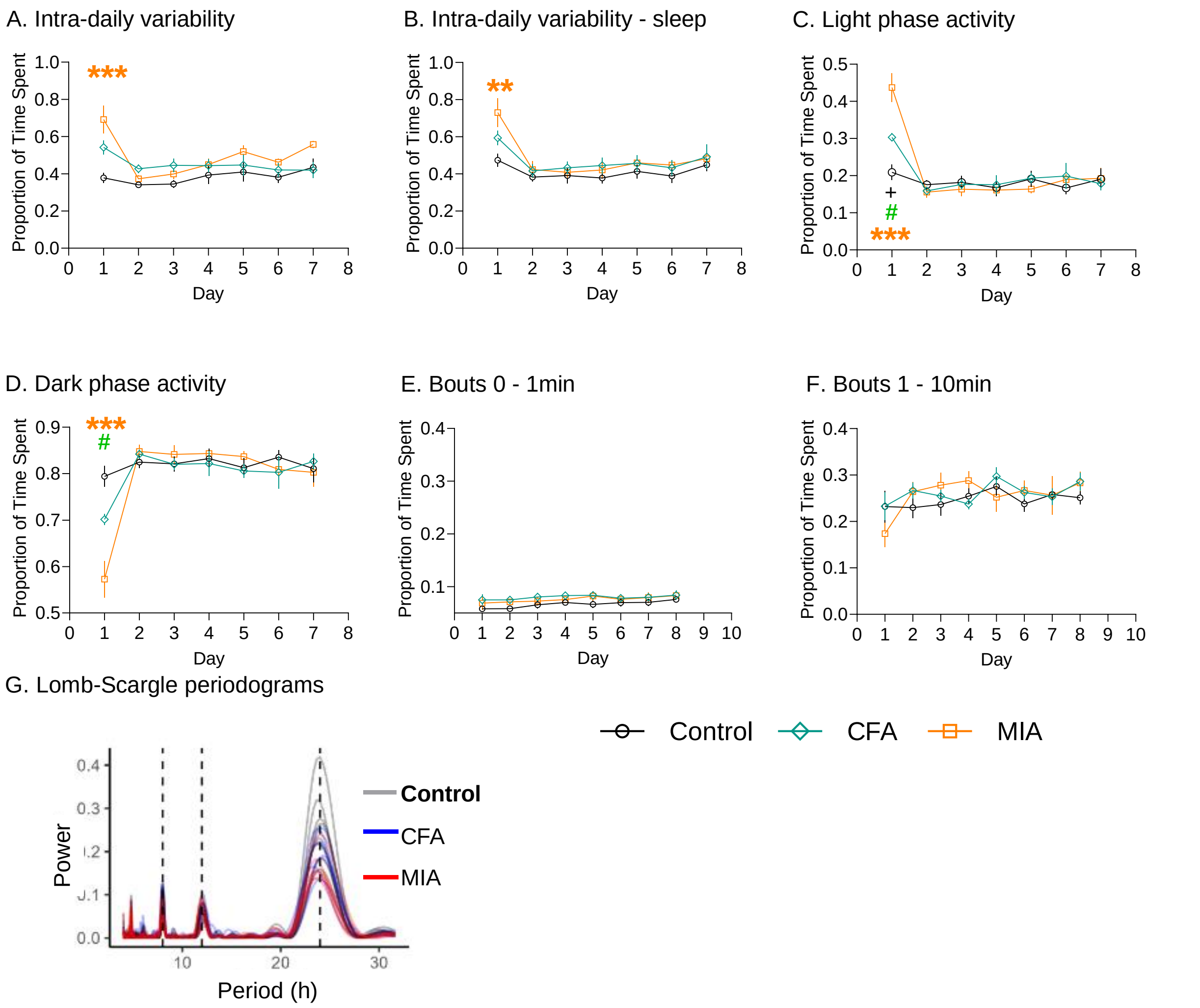


**Fig.S2: Sleep and activity patterns are more sensitive to MIA than CFA.** (**A-F**) 7-day plots using 24h bins; (**A**) Intra-daily variability. (**B**) Intra-daily variability sleep. (**C**) Proportion of activity during light period. (**D**) Proportion of activity during the dark period. (**E**) 0-1min bouts during the dark period recorded over 7 days. (**F**) 1-10min bouts during the dark period recorded over 7 days. (**G**) Lomb-Scargle periodograms for each mouse. Dashed lines mark 8, 12, and 24 hours. (**A-F**) Data shows mean ± S.E.M. N=8/8/7, Control/CFA/MIA; $^{\#}P<0.05$, CFA *vs* control; ***$P<0.001$, **$P<0.05$, MIA *vs* control, Univariate analysis at Day 1.

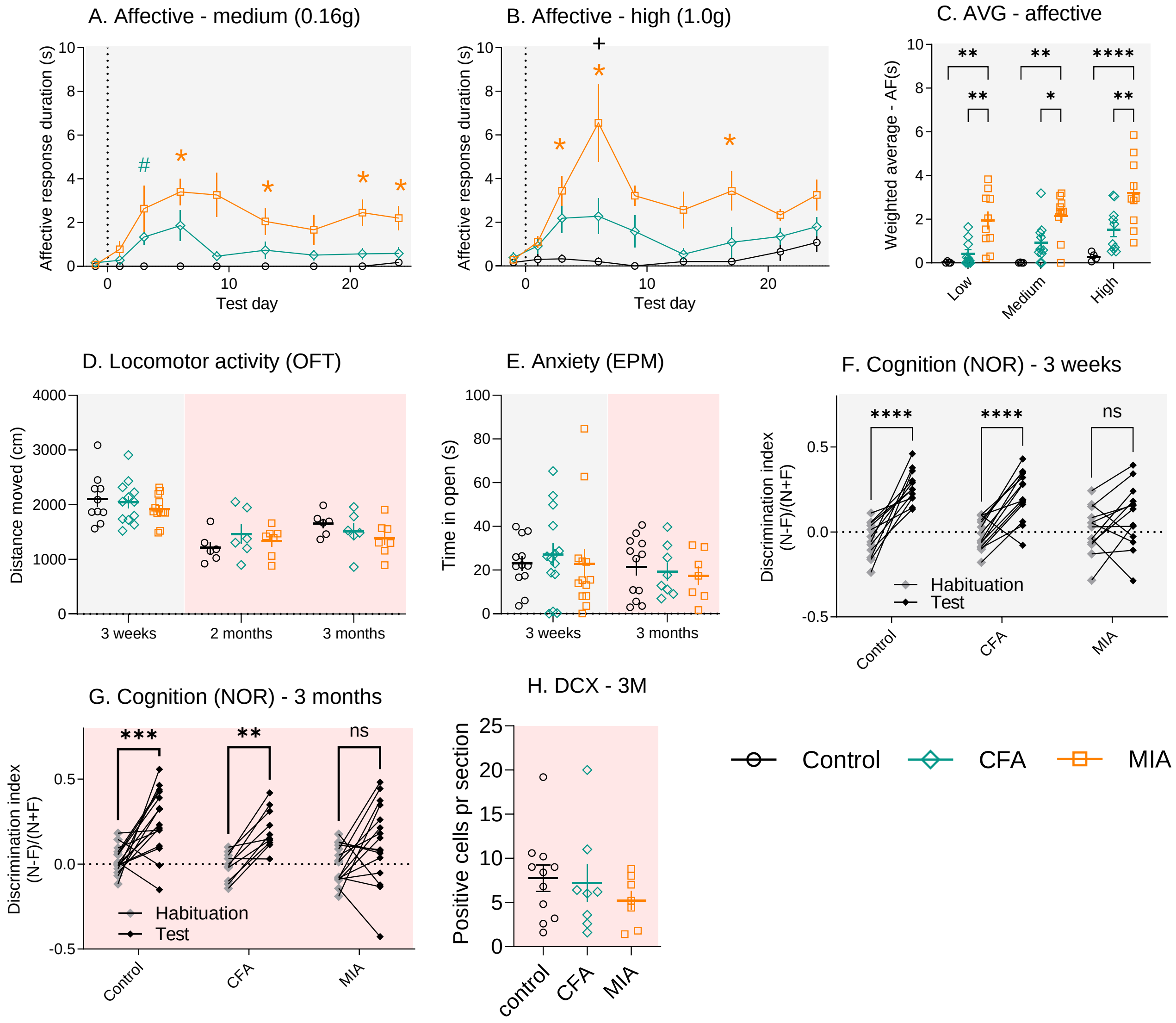


**Figure S3: The MIA but not the CFA model induces robust negative affective behaviours. (A, B)** The affective response to the application of the 0.16g and 1g Von Frey filaments was recorded in seconds (N=4-10). (**C**) Weighted average for the affective responses to all Von Frey filaments: low: 0.04g; Medium: 0.16g and High: 1g (N=4-10). (**D**) Locomotion was assessed using the distanced travelled in the Open Field test (N=6-13). (**E**) Anxiety like behaviour assessed using the amount of time exploring the open arms of the Elevated Plus Maze (N=7-14). **(F, G)** Discrimination index plots for the Novel Object Recognition test, including both habituation and test-phase (test phase displayed alone in Fig 4C. This data suggests that for the MIA-model, there is high variability in time exploring each of the two identical objects already during the familiarisation, which was unmodified by the introduction of a novel object (N=12-16). (**H**) There was no difference in expression in DCX in the hippocampus at 3 months after CFA and MIA injections. (N=7-11). Post-test in time-course figures (A, B); $^{\#}$P<0.05, CFA *vs* control; *P<0.05, MIA *vs* control, $^{+}$P<0.05 CFA *vs* MIA, as determined using Tukey's multiple comparison test. Full analysis-outcome in Supplementary table S2. For (C,G,H) *P<0.05, **P<0.01, ***P<0.001, ****P<0.0001, as determined using appropriate post-test.

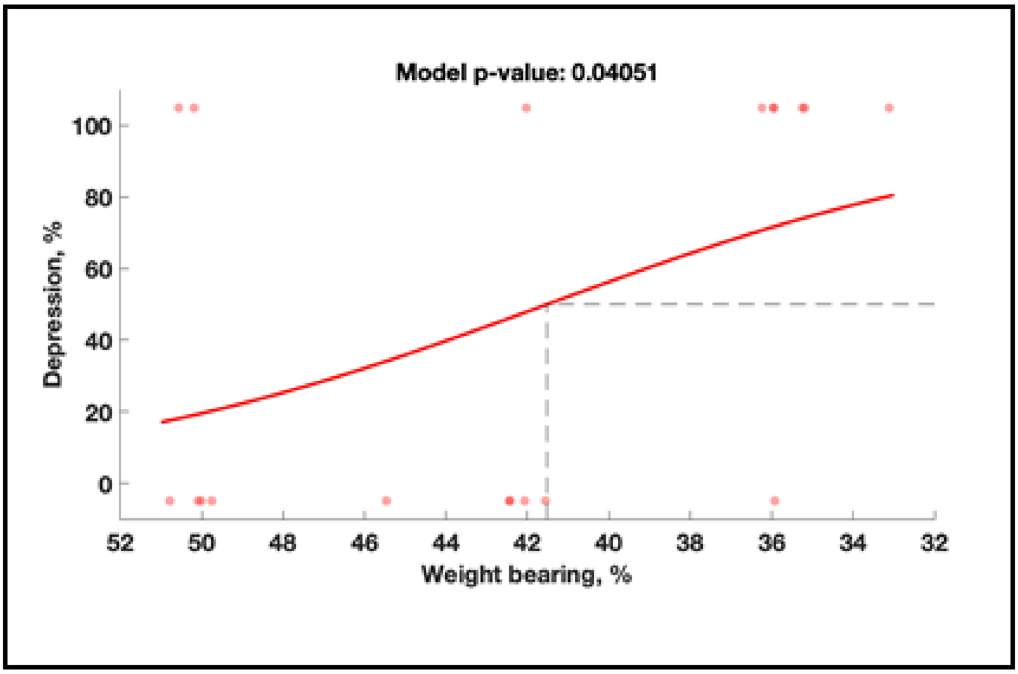


**Figure S4: Early weight bearing deficit in joint disease can be used to predict the development of depressive-like behavior in late disease stage.**

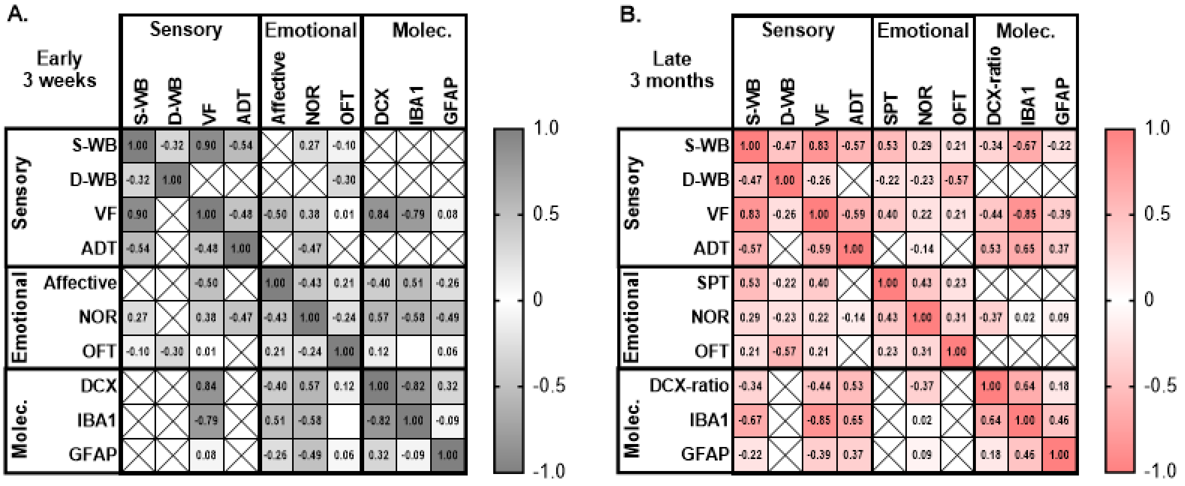


**Fig S5. Extended correlation matrix.** Summary of correlations between outcome measures recorded on the same animal at the same time point at 3 weeks (**A**) and 3 months (**B**) after injury. This extended version of figure 5 also includes the parameters, GFAP and "D-WB". Values displayed are r-values for Pearson r correlation analysis, estimating the strength of the correlations. The more intense color-coding signifies approaching the perfect fit at –1 or 1. S-WB = Static weight bearing. D-WB = Dynamic weight bearing, as assessed using the parameter, Swing Time Ratio.

**Table S1. Statistical analysis table for main figures**

| Fig | Analysis[1] | F-values | Post test |
|---|---|---|---|
| **Fig 1 – sensory changes after injury** | | | |
| Fig 1A1 – Static Weightbearing | RM ANOVA, injury*time, | $F_{injury}$ (2,16) = 324.8, P<0.0001<br>$F_{time}$ (11,176) = 20.19, P<0.0001<br>$F_{interaction}$ (2,16) = 7.863, P<0.0001 | Tukey[2]<br>D3; ***, ##, +<br>D6; ****, ####, ++<br>D8; ****, ####, +++<br>D13; **, ####, +<br>D18; ****, ##, +<br>D22; ****, ####, ++<br>D27; ***, ###, +<br>D47; ***, ####, +<br>D59; ***, ##<br>D82; ***, ##,<br>D99; ****, #, ++ |
| Fig 1A2 – WB – weighted average | RM ANOVA, injury*phase. (early vs late considered as RM) | $F_{injury}$ (2,16) = 264.5, P<0.0001<br>$F_{phase}$ (1,16) = 25.56, P=0.0001<br>$F_{interaction}$ (2,16) = 10.99, P=0.001 | Tukey<br>Results displayed in figure. |
| Fig 1B1 – Swing time ratio | RM ANOVA, injury*time, | $F_{injury}$ (2,16) = 6.311, P=0.0095<br>$F_{time}$ (11,176) = 7.308, P<0.0001<br>$F_{interaction}$ (22,176) = 3.909, P<0.0001 | Tukey[2]<br>D6; ****, ++++<br>D8; ****, ++ |
| Fig 1B2 – Swing time – weighted average | RM ANOVA, injury*phase. (early vs late considered as RM) | $F_{injury}$ (2,16) = 5.690, P=0.0136<br>$F_{phase}$ (1,16) = 6.724, P=0.0196 | Tukey<br>Results displayed in figure. |
| Fig 1C1. – Von Frey – mechanical allodynia | Mixed Effects model[1], RM, injury*time | $F_{injury}$ (2,44) = 286.9, P<0.0001<br>$F_{time}$ (17,403) = 45.98, P<0.0001<br>$F_{interaction}$ (34,403) = 13.20, P<0.0001 | Tukey[2]<br>6H; ***, ####, +<br>D1 ; ****, ####, +<br>D3; ****, ####,<br>D6; ****, ####, +<br>D9; ****, ####, ++<br>D13; ****, ####,<br>D17; ****, ####, ++ |

| | | | |
|---|---|---|---|
| | | | D21; ****, ####, ++<br>D24; ****, ####, +<br>D41; ****, ####,<br>D48; ****, ####,<br>D56; ****, ####,<br>D63; ****, ####, +<br>D71; ****, ####,<br>D78; ****, ####, +<br>D90; ****, ####, ++ |
| Fig 1C2 – VF – weighted average | RM ANOVA, injury*phase. (Early vs late considered as RM) | $F_{injury}$ (2,64) = 479.9, P<0.0001 | Tukey<br>Results displayed in figure. |
| Fig 1D1 – Acetone Drop Test – cold allodynia | Mixed Effects model[1], RM, injury*time | $F_{injury}$ (2,20) = 18.46, p<0.0001<br>$F_{time}$ (10,144) = 6.36, p<0.0001 | Tukey[2]<br>D9; ****, +<br>D17; *, ###<br>D24; ***, +<br>D73; **<br>D90; ** |
| Fig 1D2 – ADT – weighted AVG. | Mixed Effects model, RM, injury*phase (early vs late considered as RM) | $F_{injury}$ (2, 29) = 17.92, P<0.0001 | Tukey<br>Results displayed in figure. |
| Fig 1E – ankle circumference | Two-way ANOVA, time*injury | $F_{injury}$ (2,50) = 90.06, P<0.0001 | Tukey<br>Results displayed in figure. |
| Fig 1F – VF / ankle circumference correlation, CFA-injured only. | Pearson r correlation<br>Correlation of measures from early (Day 7+25) and late (3 months) | Early; r= 0.6857, P=0.0286.<br>Late; NS | |
| Fig 1G – knee circumference | Two-way ANOVA, time*injury | $F_{injury}$ (2,50) = 167.1, P<0.0001<br>$F_{time}$ (4,50) = 24.09, P<0.0001<br>$F_{interaction}$ (8.59) = 22.87, P<0.0001 | Tukey<br>Results displayed in figure. |
| Fig 1H – VF / knee circumference correlation, | Simple linear regression<br>Correlation of measures from early | NS | |

| | | | |
|---|---|---|---|
| MIA-injured only. | (Day 7+25) and late (3 months) | | |
| Fig 1I – VF/ADT correlation | Pearsons r correlation, Single timepoint correlation from 3 weeks and 3 months. | 3 weeks/early; r=-.0480, P=0.020<br>3 months/Late; r= -0.594 P=0.001. | |
| Fig 1J - VF/WB correlation | Pearsons r correlation. Single timepoint correlation from 3 weeks and 3 months. | 3 weeks / early; r=0.898, P<0.0001.<br>3 months / late; r= 0.817, P<0.0001. | |
| Fig 1K - ADT/WB correlation | Pearsons r correlation Single timepoint correlation from 3 weeks and 3 months. | 3 weeks/early; r=-0.538, P=0.008<br>3 months/Late; r= -0.570, P=0.002. | |
| Fig 1L – Swing time / WB correlation | Pearsons r correlation Single timepoint correlation from 3 weeks and 3 months. | 3 weeks/early; r=-0.555, P=0.007<br>3 months/Late; r= -0.469 P=0.021. | |
| **Fig 2 – spinal molecular changes after injury** | | | |
| Fig 2A1. C-Fos 2h | Two-way RM ANOVA, area*injury (area = L II-II, L III-V x ipsi, contra treated as repeated measure) | $F_{injury}(2,8)$ =8.2, P=0.012<br>$F_{area}(3,24)$ =23.6, P<0.001<br>$F_{interaction}(3,6)$ =4.2, P=0.029 | Tukey, Results displayed in figure. |
| Fig 2B1. CGRP – 2h | Two-way RM ANOVA, side*injury (ipsi-contra treated as repeated measure) | $F_{injury}$ (2,9) = 7.520, P=0.012 | Tukey Results displayed in figure. |
| Fig. 2C1; GFAP, I; 7 days, II; 3 weeks III; 3months | Two-way RM ANOVA, Side*injury (ipsi-contra treated as repeated measure) | D7;$F_{injury}$ (2,9) = 8.103, P=0.0097<br>$F_{side}$ (1,9) = 138.3, P<0.0001<br>$F_{interaction}$ (2,9) = 42.31, P<0.0001<br>3W; $F_{side}$ (1,13) = 11.90, P=0.0043<br>3M; $F_{side}$ (1,9) = 12.67, P=0.0061 | Tukey Results displayed in figure. |

| | | | |
|---|---|---|---|
| Fig. 2D1; IBA1,<br>I; 7 days,<br>II; 3 weeks<br>III; 3months | Two-way RM ANOVA,<br>Side*injury<br>(ipsi-contra treated as repeated measure) | D7;$F_{injury}$ (2,9) = 14.88, P=0.0014<br>$F_{side}$ (1,9) = 7.590, P=0.0223<br>$F_{interaction}$ (2,9) = 6.417, P=0.0185<br>3W;$F_{injury}$ (2,13) = 15.75, P=0.0003<br>$F_{side}$ (1,13) = 114,7, P<0.0001<br>$F_{interaction}$ (2,13) = 40.27, P<0.0001<br>3M;$F_{injury}$ (2,9) = 5.707, P=0.0251<br>$F_{side}$ (1,9) = 22.30, P=0.0011<br>$F_{interaction}$ (2,9) = 5.124, P=0.0324 | Tukey<br>Results displayed in figure. |
| Fig. 2E. IBA1/VF correlation at 7 days, 3 weeks, 3 months, | Pearson r<br>Correlation of measures from day 7, 3 weeks, 3 months | 7 days; r=-0.4194 P=0.30 (NS)<br>3 weeks/early; r=-0.7905, P=0.0003<br>3 months/Late; r= -0.8510, P=0.0004. | |
| **Fig 3. Sleep and activity pattern after injury** | | | |
| Fig 3B. Activity D1 | Two-way RM ANOVA, time*injury | $F_{time}$(23,460) =16.9, P<0.001<br>$F_{injury}$(2,20) =9.9, P<001<br>$F_{interactions}$(46,460) =3.7, P<0.001 | Tukey for Injury<br>Results displayed in figure. |
| Fig 3C. Sleep D1 | Two-way RM ANOVA, time*injury | $F_{time}$(23,460) =13.4, P<0.001<br>$F_{injury}$(2,20) =3.3, P=0.059<br>$F_{interactions}$(46,460) =3.1, P<0.001 | |
| Fig 3D. Activity D2-D4 | Two-way RM ANOVA, time*injury | $F_{time}$(23,460) =81, P<0.001<br>$F_{injury}$(2,20) =5.1, P=0.017<br>$F_{interactions}$(46,460) =1.5, P=0.128 | Tukey for Injury<br>Results displayed in figure. |
| Fig 3E. Sleep D2-D4 | Two-way RM ANOVA, time*injury | $F_{time}$(23,460) =57.9, P<0.001<br>$F_{injury}$(2,20) =8.2, P=0.002<br>$F_{interactions}$(46,460) =4.1, P<0.001 | Tukey for Injury<br>Results displayed in figure. |
| Fig 3F. Activity D5-D7 | Two-way RM ANOVA, time*injury | $F_{time}$(23,460) =67.6, P<0.001<br>$F_{injury}$(2,20) =5.1, P=0.016<br>$F_{interactions}$(46,460) =1.8, P=0.029 | Tukey for Injury<br>Results displayed in figure. |
| Fig 3G: Sleep D5-D7 | Two-way RM ANOVA, time*injury | $F_{time}$(23,460) =46.5, P<0.001<br>$F_{injury}$(2,20) =4.6, P=0.023<br>$F_{interactions}$(46,460) =3.5, P<0.001 | Tukey for Injury<br>Results displayed in figure. |

| | | | |
|---|---|---|---|
| Fig 3H: Dark phase sleep | Two-way RM ANOVA, time*injury | $F_{time}(6,120)$ =27.7, P<0.001<br>$F_{injury}(2,20)$ =8.1, P=0.003<br>$F_{interactions}(12,120)$ =3.5, P=0.012 | Tukey for Injury<br>Results displayed in figure. |
| Fig 3I: Light phase sleep | Two-way RM ANOVA, time*injury | $F_{time}(6,120)$ =28.7, P<0.001<br>$F_{injury}(2,20)$ =8.1, P=0.003<br>$F_{interactions}(12,120)$ =2.6, P=0.017 | Tukey for Injury<br>Results displayed in figure. |
| Fig 3J: Bouts 10+ min | Two-way RM ANOVA, time*injury | $F_{time}(6,120)$ =2.46, P=0.076 (NS)<br>$F_{injury}(2,20)$ =1.2, P=0.309<br>$F_{interactions}(12,120)$ =7.1, P=0.005 | Tukey for Injury<br>Results displayed in figure. |
| Fig 3K. Power | Univariate analysis | $F_{injury}(2,20)$ =5.2, P=0.015 | Tukey for Injury<br>Results displayed in figure. |
| **Fig 4 – emotional/affective comorbidities** | | | |
| Fig 4A – Affective responding – low (0.04g) | Mixed Effects model[1], RM, injury*time | $F_{injury}$ (2,21) = 15.74, P<0.0001 | Tukey[2]<br>D6; *, +<br>D21; *, + |
| Fig 4B – Anxiety (OFT) | Two-way ANOVA, time*injury | $F_{time}$ (2, 64) = 11.26, P<0.0001<br>$F_{injury}$ = NS | - |
| Fig 4C – NOR disc index. | Two-way ANOVA, injury*time (not RM) | $F_{injury}$ (2, 75) = 6.783, P=0.002 | Tukey<br>Results displayed in figure. |
| Fig 4D – NOR/VF correlation, 3W | Pearson r correlation | r=0.5279, P=0.0356 | |
| Fig 4E – DCX 3 weeks | One way ANOVA, injury | $F_{injury}$ (2, 9) = 7.990, P=0.0101 | Tukey<br>Results displayed in figure. |
| Fig 4F – NOR/DCX correlation, 3W | Pearson r correlation | r=0.5992, P=0.0391 | |
| Fig 4G – DCX 3M, left/right ratio | One-way ANOVA | $F_{injury}$ (2, 22) = 5.820, P=0.0094 | Tukey<br>Results displayed in figure. |
| Fig 4I – Anhedonia (SPT) | Two-way RM ANOVA, Time*injury | $F_{time}$ (1, 64) =0.7773, P=0.3910 = NS<br>$F_{injury}$ (2, 16) = 1.513, P=0.2501 = NS<br>$F_{time*injury}$ (2, 16) = 2.515, P=0.1122 (NS) | Tukey<br>Results displayed in figure. |

| | | |
|---|---|---|
| Fig 4J – SPT/WB correlation | Pearson r correlation at 2 and 3 months | 2 Months; P=0.35 (NS)<br>3 Months; r=0.5264, P=0.0206 |
| **Fig 5 – correlation matrix** | | |
| Fig 5A. 3 weeks / Early correlation matrix | Pearson r correlation (only correlations that are significant, or below P=0.1 are presented here) | WB/VF; r=0.898, P<0.0001.<br>WB/ADT; r=-0.538, P=0.008.<br>VF/ADT; r=-0.480, P=0.020<br>VF / affective-low; r=-0.500, P=0.049.<br>VF/NOR; r=0.385, P=0.016.<br>VF/DCX; r=0.837, P=0.0007.<br>VF/IBA-1; r=-0.791, P=0.0003.<br>ADT/NOR; r=-0.470, P=0.0236.<br>NOR/DCX; r=0.569, P=0.0395.<br>NOR/IBA-1; r=-0.584, P=0.0175.<br>DCX/IBA-1; r=-0.815, P=0.0012.<br>IBA-1/affective-low; r=0.531, P=0.042. |
| Fig 5B. 3 months / Late correlation matrix | Pearson r correlation (only correlations that are significant, or below P=0.1 are presented here) | WB/VF; r= 0.833, P<0.0001.<br>WB/ADT; r= -0.570, P=0.0019.<br>WB/NOR; r=0.288, P=0.065 (NS).<br>WB/SPT; r=0.526, P=0.0206.<br>WB/IBA-1; r= -0.668, P=0.0176<br>VF/ADT; r= -0.594, P=0.001.<br>VF/SPT; r=0.403, P=0.0869 (NS)<br>VF/DCX-ratio; r=-0.442, P=0.0446<br>VF/IBA-1; r= -0.851, P=0.0004<br>ADT/DCX-ratio; r= 0.526, P=0.0144<br>ADT/IBA-1; r= 0.648, P=0.0227<br>SPT/NOR; r=0.426, P=0.0690 (NS)<br>NOR/DCX-ratio; r=-0.367, P=0.1013 (NS)<br>DCX-ratio/IBA-1; r=0.638, P=0.0471. |
| Fig 5C. Late depressive behaviour vs early AVG static WB | Pearson r correlation | r=0.5314, P=0.0192 |
| Fig 5D. Late depressive behaviour vs early AVG dynamic weight bearing (swing ratio) | Pearson r correlation | r=-0.4892, P=0.0335 |
| Fig 5E. Late mechanical threshold vs early AVG | Pearson r correlation | r=0.8673, P<0.0001 |

| | | |
|---|---|---|
| static weight bearing | | |

[1] Mixed effects model due to different duration of experimental cohorts, meaning missing values. All cohorts though included equal numbers of animals from the different groups. Weighted average was calculated based on the period that the animals in question was tested.

[2]Post-tests for time-course figures;

- MIA vs control; *; *P<0.05, **P<0.01, ***P<0.001. ****P<0.0001.
- CFA vs control; #; #P<0.05, ##P<0.01, ###P<0.001. ####P<0.0001.
- CFA vs MIA; +; +P<0.05, ++P<0.01, +++P<0.001. ++++P<0.0001.

RM= Repeated Measures

AVG = average.

**Table S2. Statistical analysis table for main figures**

| Fig | Analysis | F-values | Post test |
|---|---|---|---|
| **Fig S1 – catwalk and bodyweight** | | | |
| Fig S1A – Duty Cycle | RM ANOVA, injury*time | $F_{injury}$ (2,16) = 4.904, P=0.0218<br>$F_{time}$ (11,176) = 11.15, P<0.0001<br>$F_{interaction}$ (22,176) = 4.129, P<0.0001 | Tukey[2]<br>D3; #<br>D6; ****, +++<br>D8; ****, ++<br>D47; + |
| Fig S1B – Stride Length | RM ANOVA, injury*time | $F_{injury}$ (2,16) = 2.853, P=0.0872 (NS) | Tukey[2]<br>D6; **, + |
| Fig S1C – Single Stance | RM ANOVA, injury*time, | $F_{injury}$ (2,16) = 1.655, P=0.2222 (NS)<br>$F_{time}$ (11,176) = 5.450, P<0.0001<br>$F_{interaction}$ (22,176) = 2.414, P=0.0008 | Tukey[2]<br>D6; **, +<br>D8; **<br>D27; *<br>D47; + |
| Fig S1D – Print position | RM ANOVA, injury*time | $F_{injury}$ (2,16) = 3.155, P=0.07 (NS)<br>$F_{time}$ (11,176) = 4.045, P<0.0001<br>$F_{interaction}$ (22,176) = 1.585, P=0.054 (NS) | Tukey[2]<br>D6; **<br>D8; **<br>D27; * |
| Fig S1H – body weight gain | RM ANOVA, injury*time, | $F_{injury}$ (2,91) = 1.151, P=0.3210 (NS)<br>$F_{time}$ (2,91) = 183.5, P<0.0001<br>$F_{interaction}$ (6,91) = 1.679, P=0.1352(NS) | |
| Fig S1E. Peak – Day 8 | Pearson r correlation matrix (only correlations that are | WB/Swing; r=-0.769, P=0.0001<br>WB/Duty cycle; r=0.846, P<0.0001<br>WB/Stride length; r=-0.362, P=0.064 (NS)<br>WB/Single stance; r=0.617, P=0.002 | |

| | | | |
|---|---|---|---|
| correlation matrix | significant, or below P=0.1 are presented) | WB/Print position; r=0.641, P=0.002<br>Single stance/print position; r= 0.338, p=0.079 (NS)<br>Single stance / swing time; r= -0.599, P=0.003<br>Single stance / duty cycle; r=0.812, P<0.0001<br>Stride length / duty cycle; r=-0.322 , P=0.089 (NS)<br>Stride length / print position; r= -0.571, P=0.005<br>Stride length / swing time; r= 0.624, P=0.002<br>Duty cycle / print position; r= 0.727, P<0.0001<br>Duty cycle / swing time; r=-0.864 . P<0.0001<br>Print position / swing time; r= -0.680, P=0.0001 | |
| Fig S1F. Early – 3W correlation matrix | Pearson r correlation matrix (only correlations that are significant, or below P=0.1 are presented) | WB/Swing; r=-0.555, P=0.007<br>WB/Duty cycle; r=0.404, P=0.043<br>WB/Single stance; r=0.354, P=0.0.069(NS)<br>WB/Print position; r=0.393, P=0.048<br>Single stance / duty cycle; r= 0.720, P<0.0001<br>Single stance / swing time; r= -0.435, P=0.031<br>Stride length / duty cycle; r= -0.533, P=0.009<br>Stride length / print position; r= -0.722, P<0.0001<br>Stride length / swing time; r= 0.687, P=0.001<br>Duty cycle / print position; r= 0.679, P=0.001<br>Duty cycle / swing time; r= -0.808, P<0.0001<br>Print position / swing time; r= -0.701, P<0.0001 | |
| Fig S1G. Late – 3M correlation matrix | Pearson r correlation matrix (only correlations that are significant, or below P=0.1 are presented) | WB/Swing; r=-0.469, P=0.021<br>WB/Duty cycle; r=0.519, P=0.011<br>WB/Single stance; r=0.338, P=0.078 (NS)<br>Single stance / stride length; r= 0.463, P=0.023<br>Single stance / duty cycle; r= 0.843, P<0.0001<br>Single stance / print position; r= -0.438, P=0.030<br>Stride length / print position; r= -0.543, P=0.008<br>Stride length / swing time; r= 0.790, P<0.0001<br>Duty cycle / swing time; r= -530, P=0.01<br>Print position / swing time; r= -0.352, P=0.07 (NS) | |
| **Fig S2 – additional sleep** | | | |
| Fig S2A. Intra – daily variability | Two-way RM ANOVA, time*injury<br>N.B. here only D1 to D6 as missing data for D7 | $F_{time}(5,100)$ =16.9, P<0.001<br>$F_{injury}(2,20)$ =5.0, P=0.017<br>$F_{interactions}(10,100)$ =3.3, P=0.008 | Tukey for Injury at D1 Results displayed in figure. |
| Fig S2B. Intra – daily variability sleep | Two-way RM ANOVA, time*injury<br>N.B. here only D1 to D6 as missing data for D7 | $F_{time}(5,100)$ =24.5, P<0.001<br>$F_{injury}(2,20)$ =1.5, P=0.246<br>$F_{interactions}(10,100)$=3.0, P=0.007 | Univariate at D1 displayed in figure. |
| Fig S2C. Light-phase activity - | Two-way RM ANOVA, time*injury | $F_{time}(6,120)$ =31.6, P<0.001<br>$F_{injury}(2,20)$ =0.852, P=0.442<br>$F_{interactions}(12,120)$ =7.6, P<0.001 | Univariate at D1 displayed in figure. |

| | | | |
|---|---|---|---|
| Fig S2D. Dark-phase activity | Two-way RM ANOVA, time*injury | $F_{time}(6,120)$ =27.3, P<0.001<br>$F_{injury}(2,20)$ =0.84, P=0.447<br>$F_{interactions}(12,120)$ =6.8, P<0.001 | Univariate at D1 displayed in figure. |
| Fig S2E. Bouts 0-1min | Two-way RM ANOVA, time*injury | $F_{time}(6,120)$ =1.6, P=0.147<br>$F_{injury}(2,20)$ =1.9, P=0.176<br>$F_{interactions}(12,120)$ =0.2, P=0.98 | |
| Fig S2F. Bouts 1-10 min | Two-way RM ANOVA, time*injury | $F_{time}(6,120)$ =3.2, P=0.026<br>$F_{injury}(2,20)$ =0.156, P=0.857<br>$F_{interactions}(12,120)$ =1.52, P=0.181 | |
| **Fig S3 – emotional supplementary** | | | |
| Fig S3A. Affective medium (0.16g) | Mixed Effects model[1], RM, injury*time. | $F_{injury}$ (2,21) = 23.47, P<0.0001 | Tukey[2]<br>D3; #<br>D6; ***<br>D13; *<br>D21; *<br>D24; * |
| Fig S3B. Affective high (1g) | Mixed Effects model[1], RM, injury*time. | $F_{injury}$ (2,21) = 11.40, P=0.0004<br>$F_{time}$ (8,128) = 3.457, P=0.0012<br>$F_{interaction}$ (8,128) = 3.457, P=0.0012 | Tukey<br>D3; *<br>D6; ****, ++++<br>D17; * |
| Fig S3C – affective weighted average | RM ANOVA, force*injury. (different filament forces considered as RM) | $F_{force}$ (2, 42) = 9.220, P=0.0005<br>$F_{injury}$ (2, 21) = 12.58, P=0.0003<br>$F_{subject}$ (21, 42) = 5.171, P<0.0001 | Tukey[2] Results displayed in figure |
| Fig S3D. Open field – distance | Two-way ANOVA, time*injury | $F_{time}$ (2,64) = 28.07, P<0.0001 | |
| Fig S3E. Elevated Plus Maze | Two-way ANOVA, time*injury | NS | |
| Fig S3F. Novel Object Recognition, 3W | RM ANOVA, injury*test-phase. | $F_{test\text{-}phase}$ (1,36) = 63.94, P<0.0001<br>$F_{interaction}$ (2,36) = 6.534, P=0.0038 | Sidak, Results displayed in figure. |
| Fig S3G. Novel Object Recognition, 3M | RM ANOVA, injury*test-phase. | $F_{test\text{-}phase}$ (1,39) = 31.70, P<0.0001<br>$F_{injury}$ (2,39) = 2.087, P=0.1376 (NS) | Sidak, Results displayed in figure. |
| Fig S3E – DCX 3M. | One-way ANOVA | P= 0.55 (NS) | |
| Fig S5A – 3 weeks. Extended | Pearson r correlation (only correlations that are significant, or | S-WB/VF; r= 0.898, P<0.0001<br>S-WB/ADT; r= -0.538, P=0.008<br>D-WB/VF; r=0.898, P<0.0001. | |

| | | |
|---|---|---|
| correlation matrix | below P=0.1 are presented here) | D-WB/ADT; r=-0.538, P=0.008.<br>VF/ADT; r=-0.480, P=0.020<br>VF / affective-low; r=-0.500, P=0.049.<br>VF/NOR; r=0.385, P=0.016.<br>VF/DCX; r=0.837, P=0.0007.<br>VF/IBA-1; r=-0.791, P=0.0003.<br>ADT/NOR; r=-0.470, P=0.0236.<br>NOR/DCX; r=0.569, P=0.0395.<br>NOR/IBA-1; r=-0.584, P=0.0175.<br>NOR/GFAP; r= -0,492, P=0.053 (NS)<br>DCX/IBA-1; r=-0.815, P=0.0012.<br>IBA-1/affective-low; r=0.531, P=0.042. |
| Fig S5B – 3months. Extended correlation matrix | Pearson r correlation (only correlations that are significant, or below P=0.1 are presented here) | S-WB/D-WB; r= -0.466, P=0.044<br>S-WB/VF; r= 0.833, P<0.0001.<br>S-WB/ADT; r= -0.570, P=0.002<br>S-WB/SPT; r= 0.526, P=0.021<br>S-WB/NOR; r= 0.288, P=0.065 (NS)<br>S-WB/IBA1; r= -0.668, P=0.018<br>D-WB/VF; r= 0.833, P<0.0001.<br>D-WB/ADT; r= -0.570, P=0.0019.<br>D-WB/NOR; r=0.288, P=0.065 (NS).<br>D-WB/SPT; r=0.526, P=0.0206.<br>D-WB/IBA-1; r= -0.668, P=0.0176<br>VF/ADT; r= -0.594, P=0.001.<br>VF/SPT; r=0.403, P=0.0869 (NS)<br>VF/DCX-ratio; r=-0.442, P=0.0446<br>VF/IBA-1; r= -0.851, P=0.0004<br>ADT/DCX-ratio; r= 0.526, P=0.0144<br>ADT/IBA-1; r= 0.648, P=0.0227<br>SPT/NOR; r=0.426, P=0.0690 (NS)<br>NOR/DCX-ratio; r=-0.367, P=0.1013 (NS)<br>DCX-ratio/IBA-1; r=0.638, P=0.0471. |

[1] Mixed effects model due to different duration of experimental cohorts, meaning missing values. All cohorts though included equal numbers of animals from the different groups. Weighted average was calculated based on the period that the animals in question was tested.

[2]Post-tests for time-course figures;

- MIA vs control; *; *P<0.05, **P<0.01, ***P<0.001. ****P<0.0001.
- CFA vs control; #; #P<0.05, ##P<0.01, ###P<0.001. ####P<0.0001.
- CFA vs MIA; +; +P<0.05, ++P<0.01, +++P<0.001. ++++P<0.0001.

RM= Repeated Measures

**Table 3.** The predictive quality of the degree of pain-burden in the first 3 weeks after injury, on the outcome at 3 months after injury. Values are Pearson r correlation analysis, and significant correlations are marked with *P<0.05: **P<0.01; ***P<0.0001. S-WB: static weight bearing; D-WB: dynamic weight bearing.

| Prediction Early vs Late | | Outcome at 3 months | | | | | | | | | |
|---|---|---|---|---|---|---|---|---|---|---|---|
| | | SPT | NOR | OFT | DCX-ratio | IBA1 | GFAP | S-WB | D-WB | VF | ADT |
| 3- week average | S-WB | 0.531* | 0.259 | 0.214 | -0.479* | -0.731** | -0.381 | 0.878*** | -0.461* | 0.889*** | -0.707*** |
| | D-WB | -0.489* | -0.066 | -0.459* | | | | -0.520* | 0.571* | -0.432 | |
| | VF | | 0.181 | | -0.357 | -0.787** | -0.376 | 0.891*** | | 0.922*** | -0.667** |
| | ADT | | -0.115 | | 0.172 | 0.671* | 0.290 | -0.842*** | | -0.870*** | 0.378 |
